# Sustained inhibition of CSF1R signaling augments antitumor immunity through inhibiting tumor-associated macrophages

**DOI:** 10.1172/jci.insight.178146

**Published:** 2025-01-09

**Authors:** Takahiko Sato, Daisuke Sugiyama, Jun Koseki, Yasuhiro Kojima, Satomi Hattori, Kazuki Sone, Hitomi Nishinakamura, Tomohiro Ishikawa, Yuichi Ishikawa, Takuma Kato, Hitoshi Kiyoi, Hiroyoshi Nishikawa

**Affiliations:** 1 Department of Immunology and; 2 Department of Hematology and Oncology, Nagoya University Graduate School of Medicine, Nagoya, Japan.; 3 Cellular and Molecular Biotechnology Research Institute, National Institute of Advanced Industrial Science and Technology (AIST), Tokyo, Japan.; 4 Laboratory of Computational Life Science, National Cancer Center, Tokyo, Japan.; 5 Department of Obstetrics and Gynecology, Nagoya University Graduate School of Medicine, Nagoya, Japan.; 6 Division of Cancer Immunology, Research Institute / Exploratory Oncology Research & Clinical Trial Center (EPOC), National Cancer Center, Tokyo, Japan.; 7 Division of Cancer Immune Multicellular System Regulation, Center for Cancer Immunotherapy and Immunobiology, Kyoto University Graduate School of Medicine, Kyoto, Japan.; 8 Kindai University Faculty of Medicine, Osaka-sayama, Japan.

**Keywords:** Immunology, Macrophages

## Abstract

Tumor-associated macrophages (TAMs) are one of the key immunosuppressive components in the tumor microenvironment (TME) and contribute to tumor development, progression, and resistance to cancer immunotherapy. Several reagents targeting TAMs have been tested in preclinical and clinical studies, but they have had limited success. Here, we show that a unique reagent, FF-10101, exhibited a sustained inhibitory effect against colony-stimulating factor 1 receptor by forming a covalent bond and reduced immunosuppressive TAMs in the TME, which led to strong antitumor immunity. In preclinical animal models, FF-10101 treatment significantly reduced immunosuppressive TAMs and increased antitumor TAMs in the TME. In addition, tumor antigen-specific CD8^+^ T cells were increased; consequently, tumor growth was significantly inhibited. Moreover, combination treatment with an anti–programmed cell death 1 (anti–PD-1) antibody and FF-10101 exhibited a far stronger antitumor effect than either treatment alone. In human cancer specimens, FF-10101 treatment reduced programmed cell death 1 ligand 1 (PD-L1) expression on TAMs, as observed in animal models. Thus, FF-10101 acts as an immunomodulatory agent that can reduce immunosuppressive TAMs and augment tumor antigen-specific T cell responses, thereby generating an immunostimulatory TME. We propose that FF-10101 is a potential candidate for successful combination cancer immunotherapy with immune checkpoint inhibitors, such as PD-1/PD-L1 blockade.

## Abstract 2

FF-10101, a unique covalently bound inhibitor of CSF1R, reduces immunosuppressive TAMs in the TME and augments antitumor immunity by enhancing tumor-specific CD8^+^ T cell responses.

## Graphical abstract

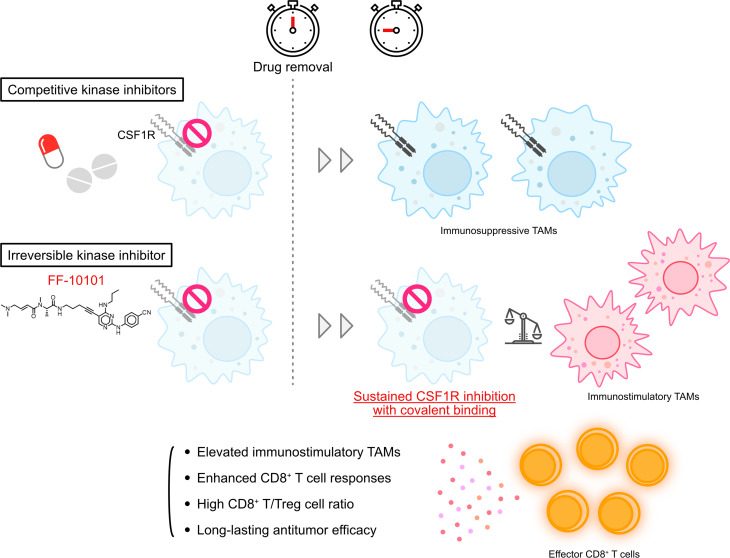

## Introduction

Cancer immunotherapy, which uses the immune system to fight cancer cells, has been successfully applied in clinical settings. The main targets of current cancer immunotherapy are coinhibitory molecules known as immune checkpoint molecules, which negatively modulate the activation of T cell responses, such as programmed cell death 1 (PD-1) and cytotoxic T lymphocyte antigen 4. Inhibitors of these molecules (immune checkpoint inhibitors, ICIs) have been approved for clinical use in various types of cancer (1). However, more than half of treated patients do not respond to ICIs; thus, the development of more effective cancer immunotherapy drugs is an urgent need.

For this purpose, resistance mechanisms to ICIs have been profoundly examined, with a particular focus on the tumor microenvironment (TME); the involvement of immunosuppressive cells, such as regulatory T (Treg) cells, myeloid-derived suppressor cells (MDSCs), and tumor-associated macrophages (TAMs), has been reported (2–4). In particular, it has been recently shown that in patients with malignant melanoma and non–small cell lung cancer, a higher gene signature of TAMs and abundant infiltration of immunosuppressive TAMs are associated with unfavorable responses to ICIs (5–8). In many tumors, M2-like macrophages constitute the majority of TAMs, which promote tumor progression by suppressing antitumor immunity; on the other hand, a high number of M1-like macrophages among TAMs has been reported to be associated with favorable patient survival (4, 9–12). In addition, immunosuppressive TAMs promote tumor invasion and migration as well as tumor angiogenesis by secreting several cytokines, chemokines, and matrix metalloproteinases. Hence, the regulation of TAMs is thought to be an important strategy to overcome resistance to ICIs. To this end, the M-CSF/colony stimulating factor 1 receptor (CSF1R) and CCL2/CCR2 pathways have been targeted to prevent the recruitment, accumulation, and polarization of macrophages into an immunosuppressive phenotype within the TME (2, 11, 12). However, the successful clinical application of CSF1R inhibitors as TAM-targeted therapies has been limited (12, 13). FF-10101 is an FMS-like tyrosine kinase 3 (FLT3) inhibitor that exhibits highly selective and covalent binding to the target (14). Based on structural similarity, it is expected to exhibit inhibitory activity against CSF1R (15). In fact, the IC_50_ against CSF1R was reported to be quite low (0.94 nM) (14). In this study, we found that FF-10101 harbored high affinity for and exerted long-lasting inhibitory activity against CSF1R, consequently controlling the immunosuppressive functions of TAMs. FF-10101 treatment increased TAMs with an M1-like phenotype. In addition, compensatory induction of tolerogenic dendritic cells (DCs) was prevented by FLT3 inhibition, a primary function of FF-10101, which overcame resistance to anti–PD-1 mAb treatment. Therefore, FF-10101 is a potential TAM-targeted therapy and could become a promising candidate for successful combination with ICIs.

## Results

### TAMs are involved in resistance to ICI therapy.

To investigate changes in the immune landscape caused by ICI therapy, we examined a publicly available dataset for single-cell RNA sequencing (scRNA-Seq) of the TME of patients with malignant melanoma who received ICI therapy (National Center for Biotechnology Information [NCBI] Gene Expression Omnibus [GEO] GSE120575) (16). We identified 22 immune cell clusters by unsupervised clustering of tumor-infiltrating CD45^+^ immune cells in the TME samples, including before and after ICI treatment (Supplemental Figure 1A; supplemental material available online with this article; https://doi.org/10.1172/jci.insight.178146DS1). Considering the expression patterns of key genes for each immune cell type, the clusters were manually annotated (Supplemental Figure 1B and Supplemental Table 1). Integrated visualization of single cells with clinical annotations according to the sampling points showed that each cluster was composed of single cells with various annotations (Supplemental Figure 1C). To identify the characteristics of ICI resistance, we performed annotation analysis with the Database for Annotation, Visualization, and Integrated Discovery (DAVID) comparing patients who did not respond with those who responded to ICI therapy with posttherapy samples. Among the differentially expressed genes, genes associated with innate immune responses were highly enriched in nonresponder patients (Supplemental Figure 1D). Gene set enrichment analysis (GSEA) further verified the significant enrichment of gene sets annotated as monocytes or macrophages in nonresponder patients (Supplemental Figure 1E).

Given the isolation of monocyte/macrophage gene sets in association with clinical responses to ICIs, the association was validated with uniform manifold approximation and projection (UMAP) visualization (Supplemental Figure 1F). The signature scores of the top 5 gene sets identified by GSEA — Monocyte:Plasma, Monocyte:Synovium, Monocyte:Undefined, M2 macrophage:Brain, and M2 macrophage:Serum — were highly expanded in the samples from nonresponder patients after ICI therapy. Furthermore, sequential biopsy of a representative nonresponder patient revealed that the accumulation of immunosuppressive macrophages occurred before Treg cell accumulation after ICI therapy (Supplemental Figure 1G), suggesting that TAMs are critical players in the formation of the immunosuppressive TME in ICI nonresponder patients.

To further validate the importance of the changes in immunosuppressive macrophage-associated gene expression in nonresponder patients, the gene expression of M2-like macrophage markers (*CD274*, programmed cell death 1 ligand 2 [*PDCD1LG2*], *CD163*, folate receptor β [*FOLR2*], and *IL10*) and *CSF1R* was examined (17–19). M2-like macrophage markers were relatively high before ICI therapy and further increased after ICI therapy in nonresponder patients (Supplemental Figure 1H). UMAP visualization revealed that these marker genes were coexpressed with *CSF1R* in clusters annotated as macrophages, especially in the cluster named 13_Immunosuppressive Macrophage (Supplemental Figure 1I). Taken together, CSF1R^+^ M2-like macrophages are associated with an unfavorable therapeutic response to ICI therapy, and CSF1R-targeted therapy may improve the therapeutic efficacy of ICI therapy.

### FF-10101 harbors long-lasting inhibitory activity against CSF1R.

**Figure 1:**
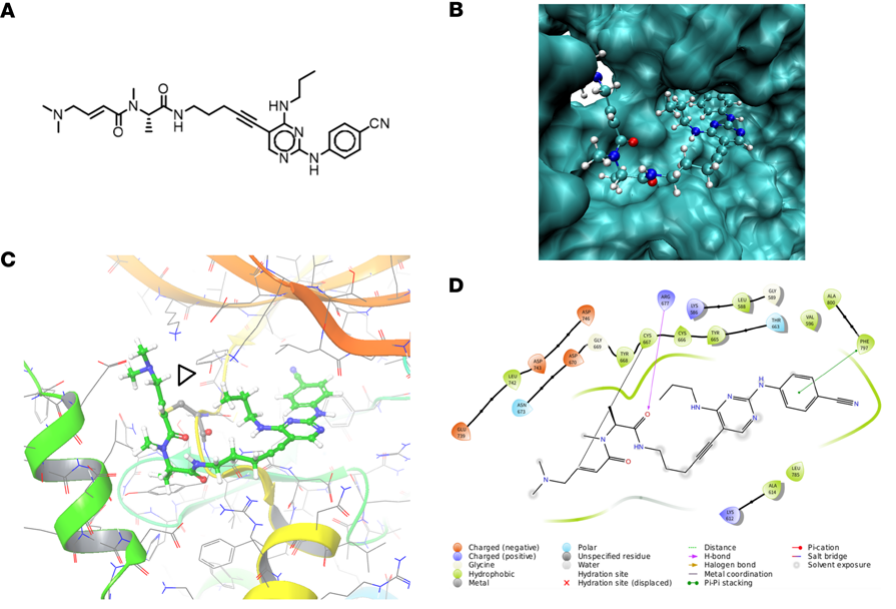
Theoretical conformational prediction reveals a stable binding of FF-10101 to CSF1R. (**A**) Chemical structure of FF-10101. (**B**) Estimated stabilized multimeric model structure of FF-10101 and CSF1R. (**C**) Predicted binding conformation between FF-10101 and CSF1R. FF-10101 is shown in ball and stick notation, and the protein structure is shown in cartoon and wire notation. The arrowhead indicates the formation of a covalent bond. (**D**) Binding interaction analysis for FF-10101 and CSF1R. The covalent bond between FF-10101 and CSF1R is shown as a black line.

FF-10101 was originally developed as an FLT3 inhibitor and strongly suppresses the proliferation of acute myeloid leukemia (AML) with *FLT3* mutations (Figure 1A) (14). Compared with other FLT3 inhibitors, FF-10101 reportedly binds to FLT3 irreversibly and exhibits long-lasting inhibitory activity by forming a covalent bond (14). In addition, FF-10101 has selective kinase inhibitory activity against type III receptor tyrosine kinases, thereby inhibiting molecules close to FLT3 in the phylogenetic tree (Supplemental Figure 2A). CSF1R, which exhibits structural similarities to FLT3 (15), was expected to be targeted by FF-10101, and FF-10101 actually showed the lowest IC_50_ among the existing CSF1R inhibitors (14, 20–23) (Supplemental Figure 2B), indicating that FF-10101 could be a good candidate for CSF1R-targeted therapy. In support of this notion, molecular docking simulations revealed that FF-10101 bound stably to dimerized FLT3 and CSF1R at low energies (ΔG_bind_ –58.71 kcal/mol and –36.46 kcal/mol, respectively) (Figure 1B). In this calculation, the covalent bond between the protein and the ligand was ignored. Moreover, the predicted positions of the binding conformations showed that the ligand–protein distance was close enough to form a covalent bond. When a covalent bond was formed on the computer, a more stable binding conformation was simulated. Therefore, it is strongly presumed that FF-10101 binds to CSF1R with a covalent bond at cysteine 667, which is homologous to cysteine 695 of FLT3 (Figure 1, C and D).

**Figure 2:**
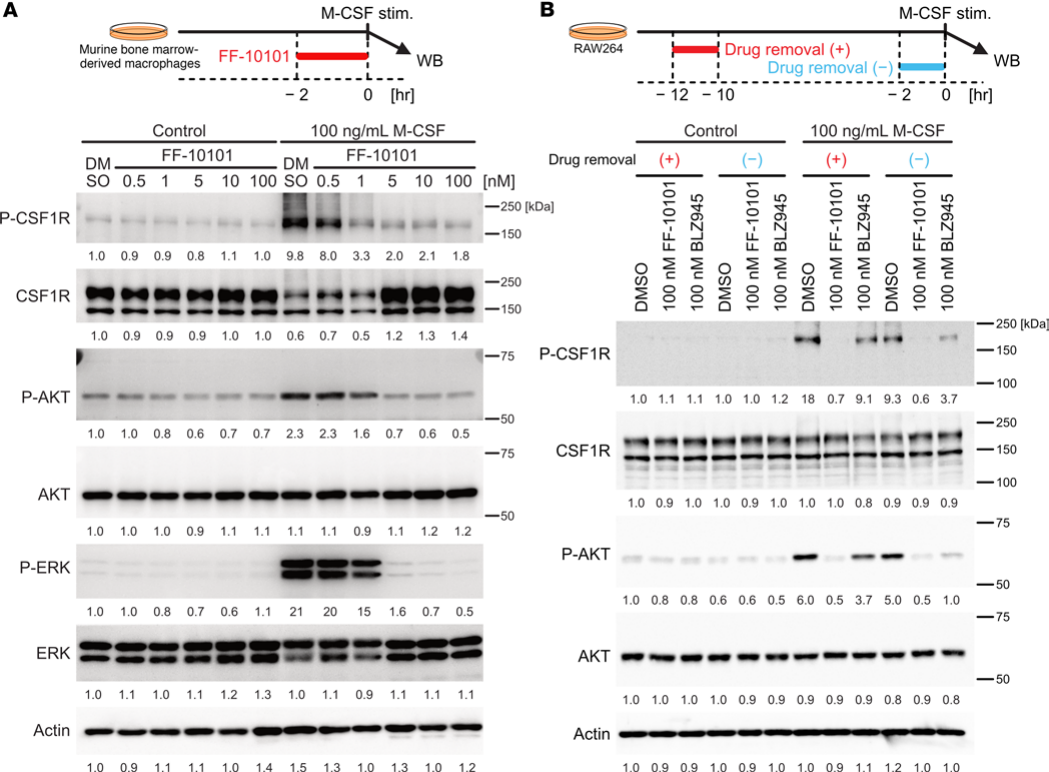
FF-10101 harbors a strong and durable inhibitory activity against CSF1R. (**A**) Western blot analyses showing the inhibition of CSF1R signaling molecules by FF-10101 treatment in murine bone marrow–derived macrophages. Murine bone marrow–derived macrophages were incubated with the indicated concentration of FF-10101. (**B**) Western blot analyses showing the persistence of CSF1R inhibitory activity of FF-10101. RAW264 cells were incubated with the indicated concentrations of FF-10101 or BLZ945. The persistence of CSF1R inhibitory activity of FF-10101 or BLZ945 was examined with (red) or without (blue) drug removal. The culture conditions are shown at the top.

To verify the inhibitory activity of FF-10101 against CSF1R, we investigated CSF1R signaling inhibition in murine bone marrow–derived macrophages (BMDMs) and human monocyte-derived macrophages induced by M-CSF, as well as in RAW264 cells, a macrophage cell line (Figure 2A and Supplemental Figure 3). The phosphorylation of CSF1R and the downstream signals, AKT and ERK1/2, in murine BMDMs and human monocyte-derived macrophages upon reexposure to M-CSF was markedly inhibited by FF-10101 in a dose-dependent manner (Figure 2A and Supplemental Figure 3A). Similar results were obtained when RAW264 cells were stimulated with M-CSF and treated with FF-10101, except for the constitutive phosphorylation of ERK (Supplemental Figure 3B). Moreover, while the inhibitory activity of BLZ945, another CSF1R inhibitor with an IC_50_ comparable to that of FF-10101, decreased upon drug removal, the activity of FF-10101 persisted even after drug removal (Figure 2B). These results suggest that FF-10101 possesses high-affinity and long-lasting inhibitory activity against CSF1R signaling and can be considered a promising candidate for CSF1R-targeted therapy.

### FF-10101 exerts antitumor efficacy by converting TAMs into M1-like macrophages.

**Figure 3:**
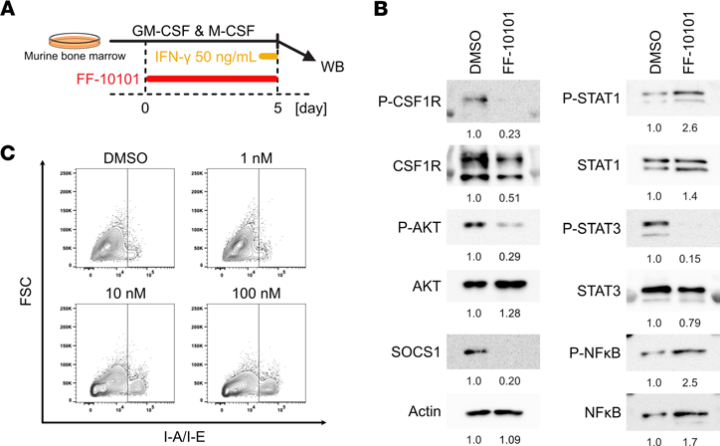
FF-10101 treatment enhances the phosphorylation of STAT1 and inhibits STAT3 signaling. (**A**) Experimental scheme. BMDMs generated with both GM-CSF and M-CSF and with or without FF-10101 were stimulated with 50 ng/mL IFN-γ for the last hour. (**B**) Western blot analyses showing the changes in protein expression between BMDMs generated with or without FF-10101 (100 nM). (**C**) Contour plots for I-A/I-E on CD45^+^CD11b^+^F4/80^+^ cells. Flow cytometry (FCM) analysis was performed 24 hours after IFN-γ stimulation.

The strong inhibitory function of FF-10101 against CSF1R signaling prompted us to examine whether FF-10101 prevented polarization to immunosuppressive M2-like macrophages, and augmented antitumor immunity, since TAMs with an M2-like phenotype attenuate antitumor immune responses and contribute to resistance to ICI therapy (4, 9, 11). We first evaluated the molecular pathways that influence the TAM phenotype in BMDMs induced by both M-CSF and GM-CSF. FF-10101 was added to the culture mixture during BMDM induction, and the BMDMs were stimulated with IFN-γ for the last hour of 5-day culture (Figure 3A). STAT1 and STAT3 signaling are known to be important for differentiation into immunostimulatory and immunosuppressive TAMs, respectively, and activation of CSF1R reportedly directly or indirectly affects their phosphorylation (24–26). FF-10101 suppressed the phosphorylation of STAT3, as well as CSF1R and AKT, and the protein expression of SOCS1 (Figure 3B). The phosphorylation of NF-κB and STAT1 was increased in the FF-10101–treated samples (Figure 3B and summarized in Supplemental Figure 4). In addition, FF-10101 addition throughout BMDM induction further enhanced the cell surface expression of MHC class II induced by IFN-γ stimulation (Figure 3C). On the other hand, FF-10101 did not affect the expression of functional molecules, such as CD86, inducible NOS (iNOS), TNF-α, or arginase-1 (Arg-1), on either preestablished M1 macrophages, i.e., BMDMs generated with GM-CSF and stimulated with IFN-γ and LPS, or preestablished M2 macrophages, i.e., BMDMs generated with M-CSF and stimulated with IL-4 and IL-13 (Supplemental Figure 5A). Thus, FF-10101 has a limited effect on the phenotype of differentiated M1 and M2 macrophages. Similarly, the expression of these functional molecules on BMDMs generated with GM-CSF did not change when they were treated with FF-10101 at the onset of stimulation with IFN-γ and LPS (Supplemental Figure 5B). On the other hand, the reduction in immunostimulatory molecules was prevented when FF-10101 was applied to BMDMs generated with M-CSF at the onset of stimulation with IL-4 and IL-13 (Supplemental Figure 5C). Furthermore, the direct impacts of FF-10101 on the proliferation of pre-established M1 and M2 macrophages were evaluated using an XTT assay. The proliferation of preestablished M1 and M2 macrophages was not affected by FF-10101 at the concentrations used for in vitro and in vivo experiments (10–100 nM) (Supplemental Figure 5D). These findings indicate that macrophages can be differentiated into immunostimulatory phenotypes in the presence of FF-10101, while FF-10101 does not alter the preestablished phenotypes of macrophages.

We then investigated whether FF-10101 inhibited tumor growth. As FF-10101 is a tyrosine kinase inhibitor, potential direct antitumor effects may compromise the interpretation of the data. Therefore, the expression of tyrosine kinases targeted by FF-10101 was tested in several tumor cell lines. Among the tumor cell lines tested, MCA205, MC38, and EMT6 lacked expression of FLT3, c-KIT, and CSF1R (Supplemental Figure 6, A–C). In addition, the direct impact of FF-10101 on cell proliferation was evaluated with an XTT assay. The proliferation of MCA205, MC38, and EMT6 cells was not affected by FF-10101, which was in sharp contrast with that of the 32D*^mtFLT3^* cell line, which stably expresses human mutant *FLT3* and is sensitive to FF-10101 (14) (Supplemental Figure 6D). These cell lines were employed for the following study. In addition, a plasma inhibitory activity (PIA) assay verified that FF-10101 orally administered through free-drinking water was properly absorbed and exhibited proper inhibitory activity in vivo (27) (Supplemental Figure 7); plasma collected from mice treated with FF-10101 (0.1 mg/mL) presented inhibitory activity equivalent to that of 10–30 nM FF-10101, which corresponded to the IC_50_ of FF-10101 against FLT3 in human plasma (28). Therefore, we employed a free-drinking model for the following in vivo experiments.

**Figure 4:**
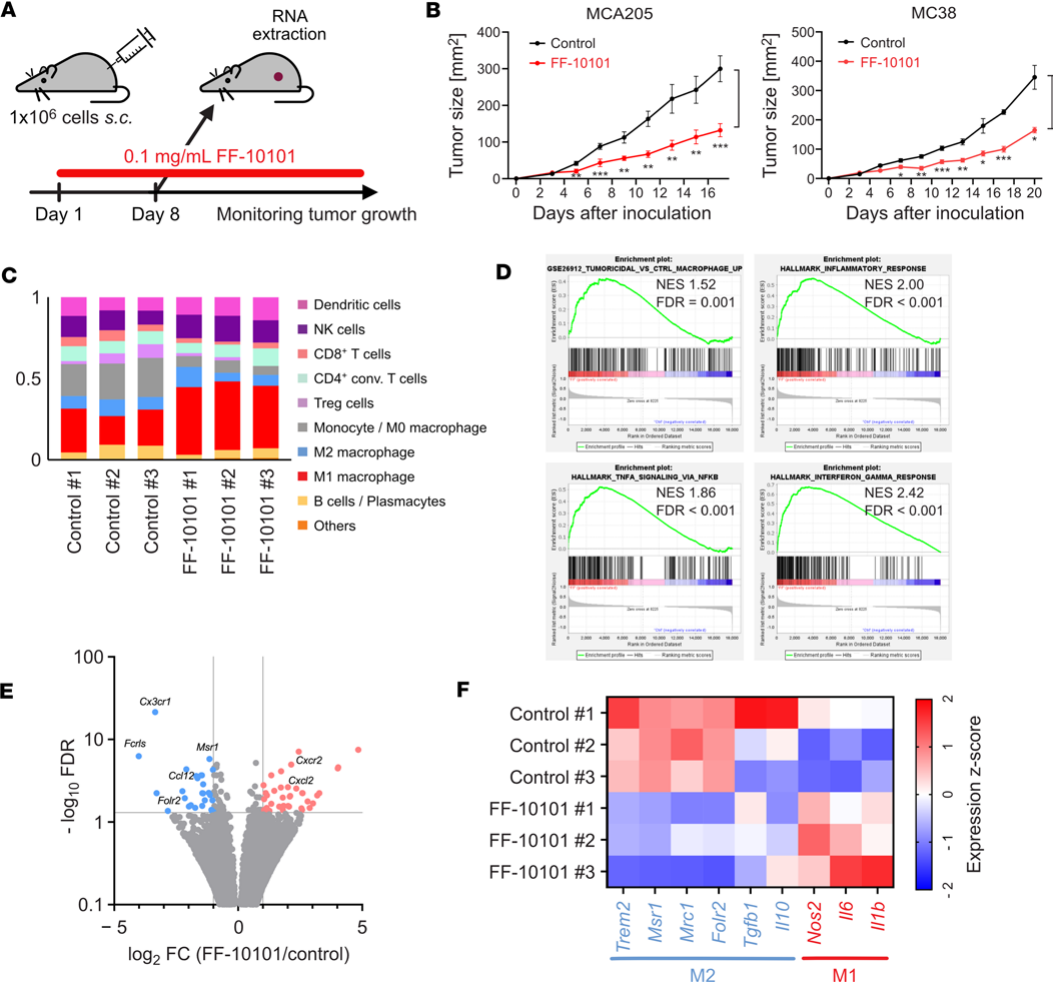
FF-10101 inhibits tumor growth by polarizing TAMs toward M1-like macrophages. (**A**) Experimental scheme. One million tumor cells (MCA205 or MC38) were inoculated into the mice on day 0, and FF-10101 was administered from day 1. (**B**) Tumor growth curves for MCA205 (left; *n* = 5 per group) and MC38 (right; *n* = 4 per group) models. The tumor volumes are shown as the means ± SDs and were compared using 2-way ANOVA with multiple *t* tests corrected with Bonferroni’s method. Adjusted *P* value: * < 0.05, ** < 0.01, *** < 0.001. (**C**–**F**) Tumors were collected on day 8 and subjected to bulk RNA-sequencing analysis (*n* = 3 per group). (**C**) Tumor bulk RNA sequencing was evaluated by CYBERSORTx. (**D**) GSEA plots of the tumoricidal macrophage, TNFα signaling, inflammatory response, and interferon-γ response gene sets for the FF-10101–treated group compared with the control group. NES, normalized enrichment score; FDR, false discovery rate. (**E**) Volcano plot of differentially expressed genes between the FF-10101 group and the control group. Molecules with significantly high and low expression in the FF-10101–treated group compared with the control group are shown in red and blue, respectively. FC, fold-change. (**F**) Heatmap of representative M1- and M2-related genes.

Mice were inoculated with MCA205 or MC38 cells and treated with FF-10101 (Figure 4A). FF-10101 significantly inhibited tumor growth in both tumor models (Figure 4B). To address the mechanism(s) of tumor regression by FF-10101, comprehensive gene expression analyses were performed with the tumor tissues collected after FF-10101 treatment. CYBERSORTx analysis using bulk RNA-sequencing data revealed that FF-10101 treatment increased the proportion of M1-like macrophages in the TME (Figure 4C). GSEA verified the marked enrichment of the gene sets associated with tumoricidal macrophage, TNFα signaling, inflammatory response, and interferon-γ response in the FF-10101–treated group (Figure 4D), suggesting that the TME was converted into a tumoricidal phenotype, such as a phenotype with high M1-like TAMs and low M2-like TAMs, by FF-10101 treatment, given that the IFN-γ response and inflammatory macrophage response have been shown to augment antitumor immune responses (29).

To further evaluate the expression of macrophage-related genes, detailed gene expression analysis was performed. M1-like macrophage-related genes (*Il1b*, *Il6*, and *Nos2*) were upregulated, whereas M2-like macrophage-related genes (*Il10*, *Tgfb1*, *Folr2*, *Mrc1*, *Msr1*, and *Trem2*) were downregulated in the TME after FF-10101 treatment (Figure 4, E and F). RNA expression was validated with quantitative real-time PCR using RNAs extracted from the TAM (CD45^+^CD11b^+^F4/80^+^) fraction in tumor tissues. The expression of M1-like macrophage-related genes, including *Il1b* and *Il6*, was increased, whereas that of M2-like macrophage-related genes, including *Cx3cr1*, *Msr1*, *Cxcl9*, and *Fcrls*, was decreased by FF-10101 in the RNA samples extracted from TAMs, as observed with bulk RNA sequencing (Supplemental Figure 8A). Given the inability of FF-10101 to repolarize M2 macrophages to M1 macrophages (Supplemental Figure 5), we argue that FF-10101 could augment antitumor immunity through the induction of M1-like macrophage differentiation from monocytes, thereby polarizing the macrophage population in the TME toward tumoricidal. Bulk RNA sequencing also revealed increases in the *Cxcl2* and *Cxcl5* expression after FF-10101 treatment (Supplemental Figure 8B).

### TAMs are reduced by FF-10101 treatment.

**Figure 5:**
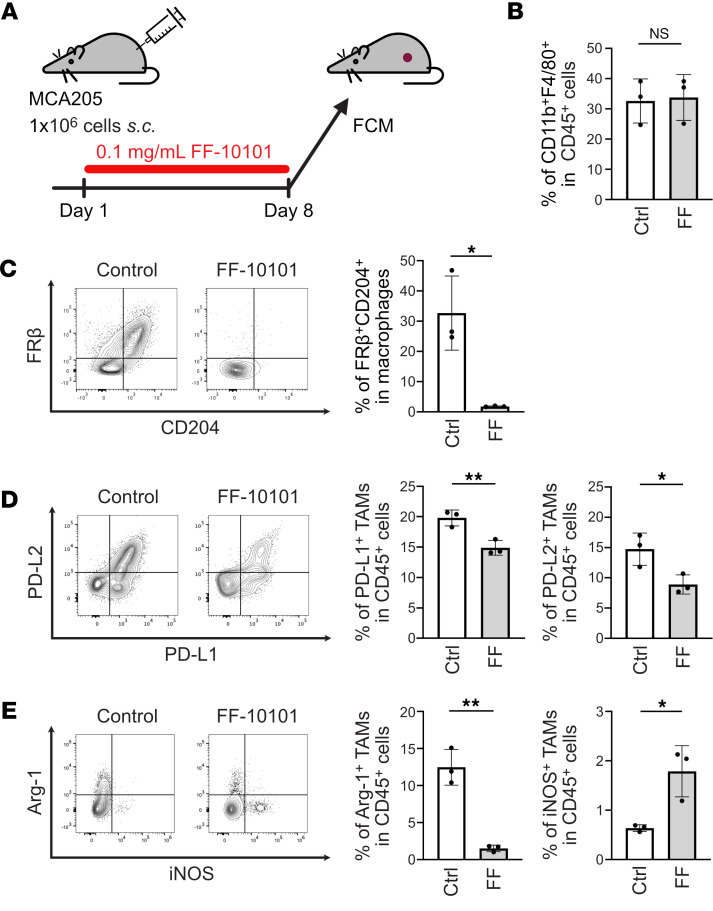
FF-10101 treatment effectively reduces immunosuppressive TAMs. (**A**) Experimental scheme. One million MCA205 cells were inoculated into the mice on day 0, and FF-10101 was administered from day 1. Immune cells were collected from tumors on day 8 and subjected to FCM analyses. (**B**) The frequency of the CD45^+^CD11b^+^F4/80^+^ fraction (TAMs) (*n* = 3 per group). (**C**) Representative contour plots of FRβ and CD204 on TAMs (left) and a summary of the frequency of FRβ^+^CD204^+^ TAMs (right; *n* = 3 per group). (**D**) Representative contour plots of PD-L1 and PD-L2 on TAMs (left) and summaries of the frequencies of PD-L1^+^ and PD-L2^+^ TAMs (right; *n* = 3 per group). (**E**) Representative contour plots of Arg-1 and iNOS on TAMs (left) and summaries of the frequencies of Arg-1^+^ and iNOS^+^ TAMs (right; *n* = 3 per group). The bar plots are shown as the means ± SDs and were compared by unpaired *t* tests. *P* values: NS ≥ 0.05, * < 0.05, ** < 0.01.

To further explore the immunological changes induced by FF-10101 treatment, immune cells in the TME were collected from MCA205 tumor–bearing mice and subjected to multicolor FCM analysis (Figure 5A). The TAM population was defined as the CD45^+^CD11b^+^F4/80^+^ population on the basis of the gating strategy shown in Supplemental Figure 9A. Initially, FCM analysis was performed on day 3, but it was difficult to perform comprehensive assessments because of the small size of the tumor masses. Therefore, we performed the following analyses on day 8, when the tumor weight had not yet differed and the TAM phenotype was similar to that on day 3 (Supplemental Figure 9, B and C). FF-10101 treatment did not affect total TAM populations but significantly reduced CD204^+^FRβ^+^ TAMs (Figure 5, B and C). In addition, the expression of programmed cell death 1 ligand 1 (PD-L1) and PD-L2 by TAMs was significantly decreased after FF-10101 treatment (Figure 5D). As CSF1R expression is reportedly high on immunosuppressive TAMs and M2 macrophages (30, 31), we examined CSF1R expression in our model. Compared with PD-L1^–^ TAMs, PD-L1^+^ TAMs presented significantly greater expression of CSF1R (Supplemental Figure 9D). These results indicate that the reduction in the expression of immune checkpoint molecules, such as PD-L1 and PD-L2, on TAMs caused by FF-10101 treatment contributes to the augmentation of antitumor immunity, as immunosuppressive M2-like phenotypes are characterized by high expression of immune checkpoint molecules (19). Moreover, Arg-1 expression by TAMs was also significantly reduced, whereas the expression of iNOS was increased after FF-10101 treatment (Figure 5E).

**Figure 6:**
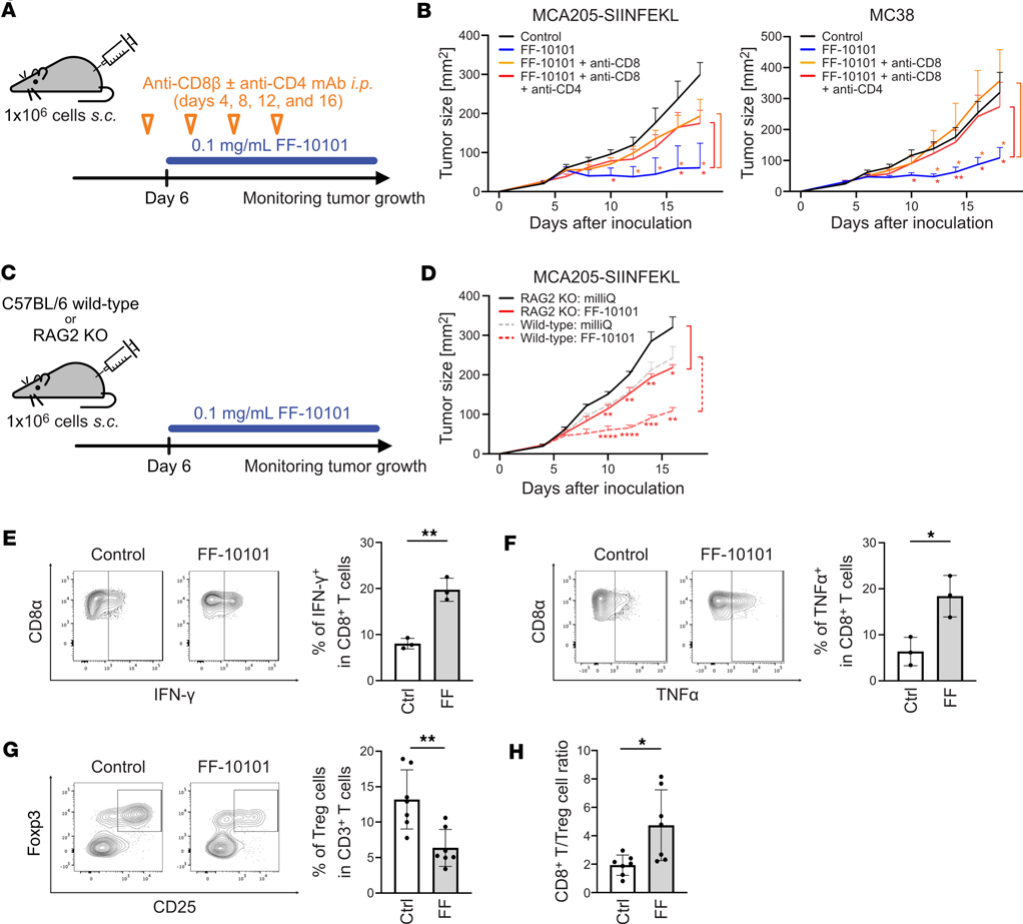
Antitumor T cell responses are induced by FF-10101 treatment. (**A**) Experimental scheme. One million tumor cells (SIINFEKL-expressing MCA205 [MCA205-SIINFEKL] or MC38) were inoculated into the mice on day 0, and FF-10101 was administered from day 6. Some mice received intraperitoneal administration of anti-CD8β mAbs and anti-CD4 mAbs on days 4, 8, 12, and 16. To examine tumor antigen-specific CD8^+^ T cell responses, MCA205-SIINFEKL was employed. (**B**) Tumor growth curves for MCA205-SIINFEKL (left) and MC38 (right) models (*n* = 5 per group). The tumor volumes between the groups were compared using 2-way ANOVA with multiple *t* tests corrected with Bonferroni’s method. Adjusted *P* values: * < 0.05, ** < 0.01. (**C**) Experimental scheme. One million tumor cells were inoculated into the mice (wild-type or RAG2 KO) on day 0, and FF-10101 was administered from day 6. (**D**) Tumor growth curves for wild-type and RAG2 KO mice (*n* = 5 per group). The tumor volumes between the groups were compared using 2-way ANOVA with multiple *t* tests corrected with Bonferroni’s method. Adjusted *P* values: * < 0.05, ** < 0.01, *** < 0.001, **** < 0.0001. (**E** and **F**) Representative contour plots (left) and a summary (right; *n* = 3 per group) of the frequency of CD8^+^ T cells producing IFN-γ (**E**) and TNF-α (**F**) analyzed as in the experimental model shown in Figure 5A. (**G**) Representative contour plots for CD25 and Foxp3 in CD4^+^ T cells (left) and a summary of the frequency of Treg cells in CD3^+^ T cells (right; *n* = 7 per group). (**H**) The ratio of CD8^+^ T cells to Treg cells in tumor tissues (*n* = 7 per group). The bar plots are shown as the means ± SDs and were compared by unpaired *t* tests. *P* values: * < 0.05, ** < 0.01.

As CD8^+^ T cells play a crucial role in killing tumor cells (32, 33), we next examined whether the antitumor efficacy of FF-10101 depended on CD8^+^ T cells. Considering the tyrosine kinase activity of FF-10101, the direct effects of FF-10101 on T cells were tested. T cell fractions, including CD8^+^ T cells, conventional CD4^+^ T (T_conv_: CD4^+^CD25^–^) cells, and Treg (CD4^+^CD25^+^) cells, were prepared from spleens and stimulated with anti-CD3 mAb and anti-CD28 mAb for 48 hours. The proliferation and viability of each T cell fraction were comparable with or without FF-10101 treatment (Supplemental Figure 10, A and B), indicating that the influences on T cell populations are not direct effects of FF-10101 but rather secondary effects caused by the conversion of TAMs. Then, the mice were inoculated with MCA205 or MC38 cells, and effector T cells, such as CD4^+^ T cells or CD8^+^ T cells, were depleted with anti-CD4 mAb and anti-CD8β mAb (Figure 6A). The antitumor effect of FF-10101 treatment was abrogated by CD8^+^ T cell depletion, and additional CD4^+^ T cell depletion did not further reduce the effect of CD8^+^ T cell depletion (Figure 6B). We also verified the antitumor effect of FF-10101 in B6.Cg-*Rag2^tm1.1Cgn^*/J (RAG2-knockout, RAG2-KO) mice (Figure 6C); FF-10101 failed to show antitumor effects on RAG2-KO mice compared with wild-type mice, indicating that the antitumor effect of FF-10101 is mediated mainly by effector CD8^+^ T cells (Figure 6D). In line with this, CD8^+^ T cells producing IFN-γ and TNF-α were significantly increased after FF-10101 treatment (Figure 6, E and F). In addition, as Treg cells decreased, the CD8^+^ T cell/Treg cell ratio, which reportedly corresponds to antitumor activity (32), significantly increased after FF-10101 treatment (Figure 6, G and H). As expected from the bulk RNA-sequencing data showing an increase in *Cxcl2* and *Cxcl5* expression after FF-10101 treatment (Supplemental Figure 8B), there was a trend toward an increasing frequency of PMN-MDSC (CD45^+^CD11b^+^F4/80^–^Ly6G^+^Ly6C^–^) population in the TME after FF-10101 treatment; however, the extent of the PMN-MDSC population varied in mice bearing different tumor cell lines (Supplemental Figure 11A). Moreover, FF-10101 was effective even in the EMT6 model, which harbored significantly high infiltration of PMN-MDSCs in the TME. On the other hand, there were no significant changes in the populations of monocytic MDSCs or conventional DCs; however, the percentages of CD11c^+^I-A/I-E^+^BST2^+^ DCs (plasmacytoid DCs: pDCs) were significantly reduced in the TME after FF-10101 treatment (Supplemental Figure 11B and 12A). In addition, FF-10101 treatment significantly reduced the percentage of PD-L1^+^PD-L2^+^ DCs in the tumor-infiltrating immune cells (Supplemental Figure 12, B and C). Taken together, FF-10101 treatment augments antitumor efficacy mainly by enhancing CD8^+^ T cell responses by reducing immunosuppressive TAMs.

### FF-10101 exhibits a long-lasting effect.

**Figure 7:**
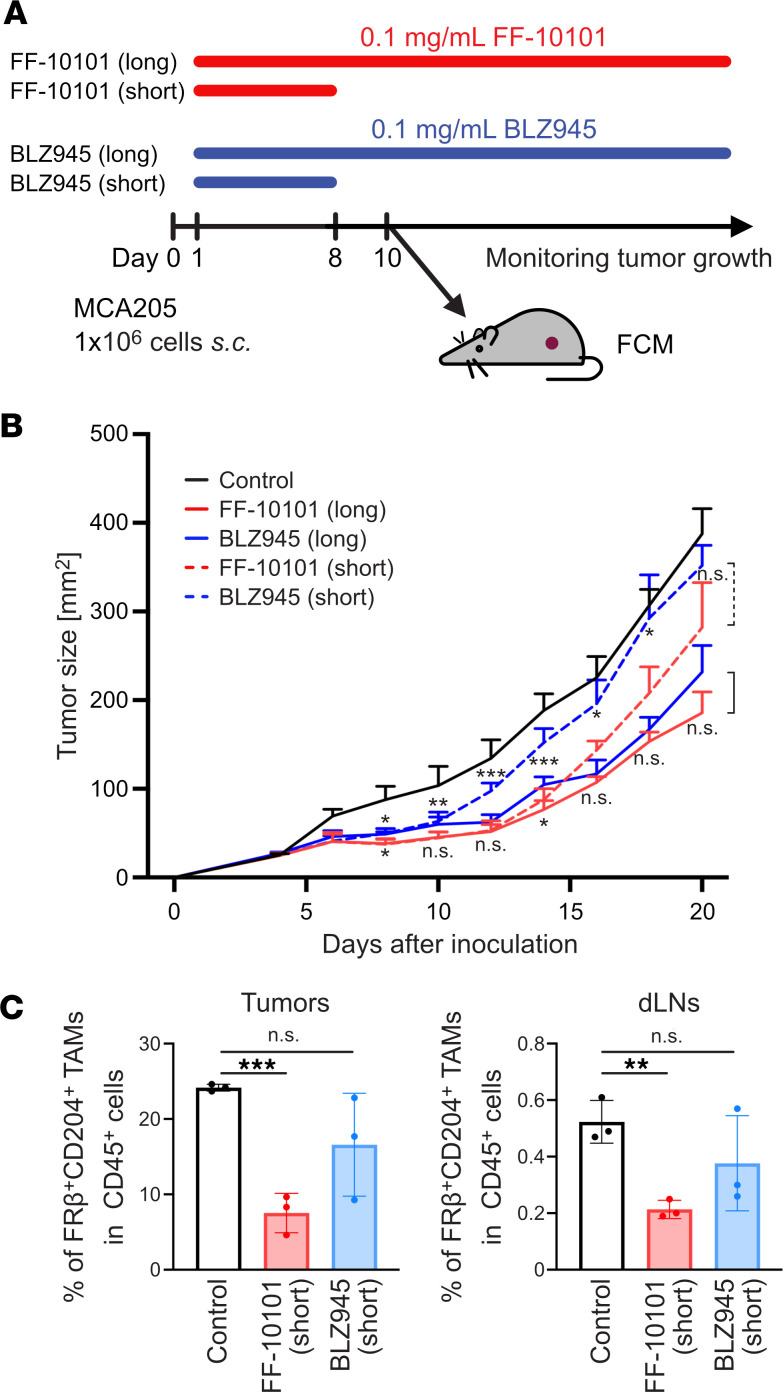
FF-10101 exhibits a long-lasting antitumor effect in vivo. (**A**) Experimental scheme. One million MCA205 cells were subcutaneously inoculated into the mice on day 0, and drugs (FF-10101 or BLZ945) were administered from day 1. In a short-term study, FF-10101 or BLZ945 was only administered from day 1 to day 8. Tumor tissues and draining lymph nodes (dLNs) were extracted on day 10 and subjected to FCM analyses. (**B**) Tumor growth curves for long-term groups (solid lines) and short-term groups (dashed line) (*n* = 10 per group). The tumor volumes between the groups were compared using 2-way ANOVA with multiple *t* tests corrected with Bonferroni’s method. Adjusted *P* values: NS ≥ 0.05, * < 0.05, ** < 0.01, *** < 0.001. (**C**) Summaries of the frequency of FRβ^+^CD204^+^ TAMs in CD45^+^ cells (*n* = 3 per group). The data are shown as the means ± SDs and were compared by unpaired *t* test. *P* values: NS ≥ 0.05, ** < 0.01, *** < 0.001.

Given the long-lasting inhibitory effect of FF-10101, as shown in Figure 2B, we compared the antitumor effects between short-term (short) and long-term (long) administration of FF-10101 (Figure 7A). The long-term administration of BLZ945 (0.1 mg/mL), a control CSF1R inhibitor, exhibited tumor growth inhibition comparably to FF-10101 (0.1 mg/mL) (Figure 7B). In sharp contrast, short-term administration of FF-10101 inhibited tumor growth similar to that of long-term administration of FF-10101, whereas the antitumor activity was rapidly abolished after short-term administration of BLZ945 (Figure 7B). Accordingly, a significant reduction of FRβ^+^CD204^+^ TAMs was observed even after drug removal in the FF-10101–treated mice but not in the BLZ945-treated mice (Figure 7C). These data indicate that FF-10101 treatment persistently converts the TME into a pro-inflammatory M1-like macrophage-rich microenvironment, which leads to a high CD8^+^ T/Treg cell ratio by continuously blocking polarization toward M2-like macrophages.

### Combination treatment with FF-10101 and anti–PD-1 mAb exhibits a far stronger antitumor effect than either treatment alone.

**Figure 8:**
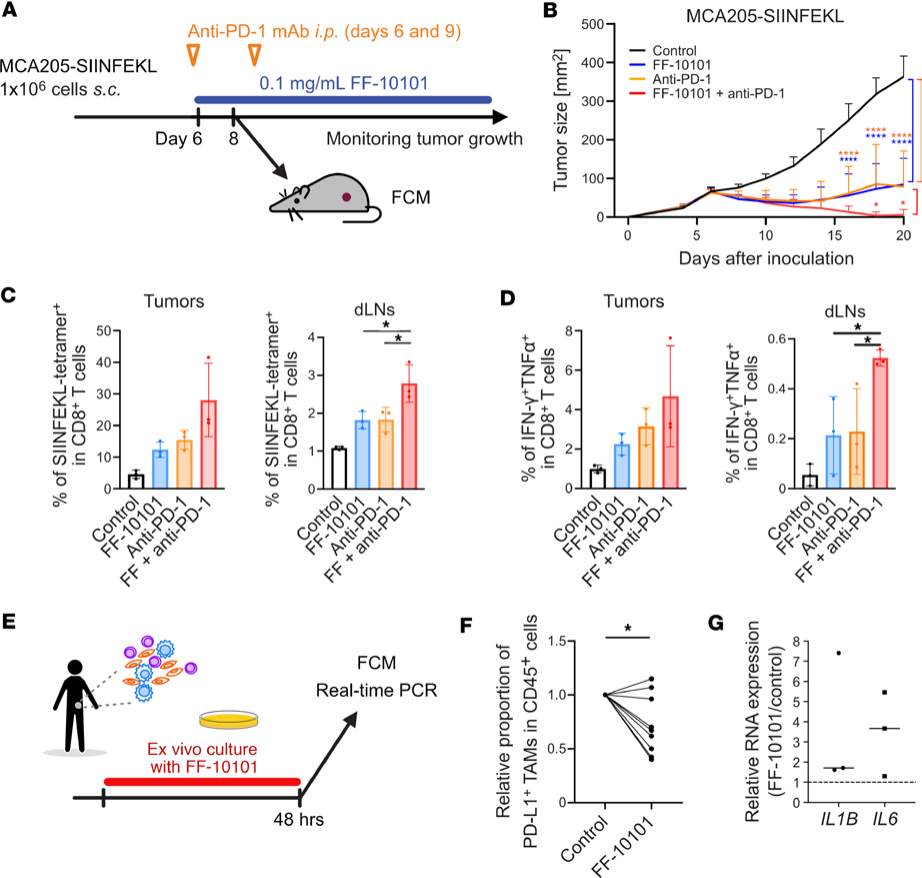
FF-10101 treatment exhibits antitumor activity by targeting TAMs in mice and humans. (**A**) Experimental scheme. One million MCA205-SIINFEKL cells were inoculated into the mice on day 0. Some mice received FF-10101 treatment from day 6 and/or anti–PD-1 mAb treatment on days 6 and 9. (**B**) Tumor growth curves for control mice and mice that received FF-10101 treatment and/or anti–PD-1 mAb treatment (*n* = 12 per group). Comparisons between 2 groups were conducted by 2-way ANOVA with multiple *t* tests corrected with Bonferroni’s method. Adjusted *P* values: * < 0.05, **** < 0.0001. (**C**) Summaries of the frequency of tumor antigen-specific (SIINFEKL-tetramer^+^) CD8^+^ T cells in tumor tissues (left; *n* = 3 per group) and dLNs (right; *n* = 3 per group). (**D**) Summaries of the frequency of IFN-γ^+^TNF-α^+^CD8^+^ T cells in tumor tissues (left; *n* = 3 per group) and dLNs (right; *n* = 3 per group). The data are shown as the means ± SDs and were compared by unpaired *t* test. *P* values: * < 0.05. (**E**) Experimental scheme. The cells were extracted from primary tumor specimens and cultured with or without 10 nM FF-10101. (**F**) Reduction rates of the PD-L1^+^ TAMs (CD3^–^CD11b^+^CD14^+^) in CD45^+^ cells (*n* = 9). Statistical analysis by paired *t* test; * *P* < 0.05. (**G**) Relative RNA expression of representative M1-related genes in TAMs was assessed by quantitative real-time PCR (*n* = 3).

The strong inhibitory effect of FF-10101 treatment against immunosuppressive TAMs led us to explore the potential of combination treatment with FF-10101 and an anti–PD-1 mAb. TAMs reportedly compromise the antitumor effect of PD-1 blockade (8–11, 34). Tumor antigen-specific CD8^+^ T cells activated by PD-1 blockade induce M-CSF production by tumor cells, which leads to TAMs’ accumulation in the TME (6). Mice were inoculated with MCA205 cells expressing SIINFEKL (a model antigen derived from ovalbumin) and treated with FF-10101, anti–PD-1 mAb, or the combination of FF-10101 and anti–PD-1 mAb (Figure 8A). Treatment with FF-10101 or anti–PD-1 mAb significantly inhibited tumor growth (Figure 8B and Supplemental Figure 13). Combination treatment exhibited a far stronger antitumor effect than either treatment alone or the control (Figure 8B and Supplemental Figure 13). Accordingly, the combination treatment increased tumor antigen-specific CD8^+^ T cells (Figure 8C), which produce multiple effector cytokines, such as IFN-γ and TNF-α (Figure 8D), in the dLNs and the TME. The combination treatment efficacy of FF-10101 and anti–PD-1 mAb was also verified in the EMT6 model (Supplemental Figure 14), which is known to be refractory to PD-1/PD-L1 blockade and exhibits an immune-excluded phenotype (35, 36). Therefore, FF-10101 is a promising candidate for combination with PD-1 blockade therapy.

### FF-10101 alters TAMs into M1-like macrophages in human tumors.

We further evaluated the activity of FF-10101 on TAMs using human tumor specimens. Immune cells were isolated from surgically resected primary endometrial cancer specimens and cultured with or without FF-10101 for 48 hours (Figure 8E). FF-10101 significantly reduced TAMs expressing PD-L1 (Figure 8F). Furthermore, the gene expression associated with M1-like macrophages (*IL1B* and *IL6*) was increased with FF-10101 treatment (Figure 8G), suggesting that FF-10101 could increase immunostimulatory TAMs in humans, as observed in animal models.

## Discussion

Despite the clinical success of cancer immunotherapy, particularly PD-1 blockade therapy, some patients experience resistance to the therapy (33). Accumulating evidence has revealed that immunosuppressive TAMs are a critical component of resistance to PD-1 blockade therapy (5, 8, 33). Macrophages play dual roles in tumor immunity. M1-like macrophages inhibit tumor growth; in contrast, M2-like macrophages, so-called immunosuppressive TAMs, play pro-tumoral roles by supporting tumor cell growth, migration, and invasion via the suppression of effector T cells, including CD8^+^ T cells (9, 11). Hence, inhibition of the recruitment and polarization of immunosuppressive TAMs could be a potential option to augment antitumor immune responses. While the M-CSF/CSF1R axis is a promising therapeutic target (12), reagents targeting this pathway have not been successfully applied in the clinic. While clinical trials of small molecule CSF1R inhibitors and CSF1/CSF1R antibodies have been conducted in various types of cancer, a single reagent, PLX3397, has been clinically approved only for tenosynovial giant cell tumor (TGCT) (13, 37). TGCT is unique because the tumor itself is dependent on CSF1R signaling (38). Suppression of TAMs by CSF1R inhibition alone could not universally and continuously show antitumor effects (13). Therefore, rather than monotherapy, combination therapies, especially those with ICIs, have been actively investigated in clinical trials (summarized in refs. 13, 37). The reagents currently being tested in clinical trials are small molecules with competitive inhibitory activity and anti-CSF1R antibodies, and little consideration has been given to the persistence of drug binding. Here, we demonstrated that FF-10101, a high-affinity and covalently bound inhibitor of CSF1R, exhibited a strong and sustained antitumor effect by inducing a tumoricidal M1-like macrophage-rich TME, which led to enhanced tumor-specific CD8^+^ T cell responses.

Long-lasting inhibition of CSF1R by FF-10101 can continuously prevent the recruitment and polarization of immunosuppressive TAMs in the TME; however, compensatory induction of M-CSF and the recruitment of other immunosuppressive cells, such as PMN-MDSCs and cancer-associated fibroblasts (CAFs), reportedly occur in a secondary resistance mechanism after CSF1R inhibition (39, 40). In our study, RNA sequence analysis revealed an increase in *Cxcl2* and *Cxcl5,* which tended to be associated with an increase in PMN-MDSCs after FF-10101 treatment. However, the extent of the increase in PMN-MDSCs in the TME varied depending on the tumor cell line, and no correlation with treatment efficacy was observed. These results are consistent with previous reports showing that the compensatory increase in PMN-MDSCs after CSF1R blockade is not universally observed in all tumor types (41). Although the increased secretion of *Cxcl1* or *CXCL8* by CAFs induced by CSF1R blockade has been reported as one of the mechanisms contributing to the accumulation of PMN-MDSCs, the heterogeneity of the TME may affect the accumulation of PMN-MDSCs and contribute to the different outcomes (39, 41). While the mechanism and functional significance remain unclear, FF-10101 appeared to increase CSF1R protein expression in M-CSF–induced mouse and human macrophages. Some DC populations are induced from monocytes through M-CSF and FLT3 ligand stimulation and play important roles in effector T cell activation. Given that FF-10101 is an FLT3 inhibitor, it may also compromise the differentiation of DCs with anti- and pro-tumoral functions. Indeed, pDCs, which are strictly dependent on FLT3 signaling for their development and compromise antitumor immunity as tolerogenic cells in the TME (42–45), were decreased in the TME after FF-10101 treatment in our animal models. Moreover, FF-10101 treatment significantly reduced PD-L1^+^PD-L2^+^ DCs in the TME (Supplemental Figure 12, B and C). Double inhibition against CSF1R and FLT3 can provide dual value for favorable outcomes in our model.

In addition to TAMs, Treg cells are another critical factor for resistance to PD-1 blockade (46, 47). Since PD-1 blockade unexpectedly activates Treg cells with PD-1 expression, modulating Treg cells in the TME is also important for successful cancer immunotherapy (33). While we did not observe a direct effect of FF-10101 treatment on Treg cells, Treg cells in the TME were significantly reduced after FF-10101 treatment, resulting in a high CD8^+^ T cell/Treg cell ratio. It is plausible that changes in the TME toward immunostimulatory conditions, such as the induction of the IFN-γ response, may have prevented the accumulation of Treg cells in the TME. In line with these findings, combination treatment with FF-10101 and anti–PD-1 mAb exhibited a far stronger antitumor effect compared to either treatment alone, with an increased CD8^+^ T cell/Treg cell ratio. Therefore, FF-10101 can make an optimal flow of antitumor immunity from innate immunity (M1-like macrophages) to acquired immunity (CD8^+^ T cells).

When a CSF1R inhibitor is considered as a combination therapy candidate for ICIs, particularly PD-1 blockade, in refractory patients, we argue that stratification of patients on the basis of the TME of each patient would be necessary. In the case of FF-10101, patients harboring abundant immunosuppressive TAMs in the TME could become an optimal target population. For clinical immunomonitoring of the TME, we have already established a clinically feasible method to analyze immune cell profiles from small clinical specimens (33, 48). In addition, while tumor *P53* mutations are known to lead to TAM accumulation, activation of KRAS signaling leads to PMN-MDSC accumulation, and *EGFR* mutations lead to Treg cell activation (49–52). Therefore, incorporating information on gene alterations in cancer cells into our immunomonitoring method would help in the consideration of appropriate combination drugs for ICI therapy.

In conclusion, FF-10101 could be a potent immunomodulatory agent that can reduce immunosuppressive TAMs in the TME via continuous inhibition of CSF1R. Given the key role of TAMs in the resistance to PD-1 blockade, FF-10101 is a promising partner in combination therapy. Appropriate clinical immunomonitoring combined with genomic analysis of cancer cells enables the identification of patients in whom combination therapy with FF-10101 and PD-1 blockade will provide robust antitumor effects.

## Methods

### Sex as a biological variable.

Our study exclusively examined female mice, except for the experiment with B6.Cg-*Rag2^tm1.1Cgn^*/J mice. It is unknown whether the results are relevant to male mice. As for the ex vivo human cell culture, all patients were female because primary endometrial cancer tissues were used in this study.

### scRNA-Seq data processing.

Single-cell gene expression data quantified as transcripts per million were downloaded from the GEO (accession number GSE120575) (16). Scanpy version 1.8.2 and AnnData version 0.7.8 were used for data processing and visualization following their documentation (53). Dimension reduction was conducted using principal component analysis of highly variable genes, and visualization was performed by UMAP. Clustering was performed with the Leiden algorithm (54) and annotated manually based on their specific marker genes (Supplemental Table 1). Genes whose expression was significantly increased in nonresponder patients (*P* < 0.01) were explored with DAVID analysis (55). GSEA was performed with GSEApy version 0.10.8 (56).

### Compounds.

FF-10101 was synthesized by the FUJIFILM Corporation as described previously (14). The salt of succinate FF-10101 (FF-10101-01) was provided by FUJIFILM Corporation. For in vitro and ex vivo assays, FF-10101-01 was dissolved in DMSO at 20 mM and stored at –30°C. FF-10101 was diluted in water to obtain 0.1 mg/mL FF-10101, which was subsequently supplied to the mice. The kinase inhibitory activity of FF-10101 was plotted with KinMap (14, 57). BLZ945 was obtained from MedChemExpress (catalog HY-12768). BLZ945 was dissolved in DMSO at 100 mg/mL and stored at –30°C. BLZ945 was diluted in water containing 0.1% 2-hydroxypropyl-beta-cyclodextrin (FUJIFILM Wako Pure Chemical; catalog 324-84233). Vehicle controls in cell culture contained less than 0.001% DMSO, except for the XTT assay as described below.

### Theoretical conformational predictions and analyses.

To predict the high and stable binding mode of FF-10101, the potential complex structure in vivo was predicted and applied for theoretical structural analysis. FGFR2 (PDB ID: 2PVY) (58), which has high amino acid sequence homology with both FLT3 and CSF1R and a sufficiently small root mean square deviation of the monomer crystal structure, was used for the template structure for multimeric structure prediction. The Homology Modeling method was applied to predict the stabilized multimeric model structure in vivo. The 3-dimensional structure of CSF1R was predicted using AlphaFold3 (59) based on the reference sequence (NP_005202.2).

For the predicted FLT3 and CSF1R complexes, docking simulations were performed to explore the FF-10101 binding complex structure. In this calculation, the covalent bond between the protein and the ligand was ignored, and the dangling-bond ends were capped with hydrogen. All docking simulations were performed using Glide of the Schrödinger suite. To confirm the validity of the ligand binding conformation even in the absence of covalent binding, the binding conformation of FF-10101 to FLT3 was explored a priori, and no significant difference was detected between the predicted optimal conformation and the experimentally observed ligand conformation (PDB ID: 5X02) (data not shown) (14).

For the optimal binding configurations obtained, the orientation and positional relationship of the target residues were checked to determine if covalent bonds could be formed. The structure was then relaxed by forming a covalent bond between the protein and the ligand and performing energy minimization with the structure as the initial structure. Interaction analysis between the ligand and protein was performed on the stabilized structures using the Schrödinger suite.

### Cell lines.

MC38, a mouse colon cancer cell line of C57BL/6 origin, was purchased from Kerafast (catalog ENH204). EMT6, a murine breast cancer cell line of BALB/c origin, was purchased from ATCC (catalog CRL-2755). MCA205, a murine cell line derived from a 3-methylcholanthrene-induced fibrosarcoma in C57BL/6 mice, was obtained from Merck (catalog SCC173). MCA205 cells stably expressing ovalbumin-derived H-2K^b^ binding peptide (SIINFEKL) were established as previously described (60). RAW264, a murine macrophage cell line, was purchased from RIKEN cell bank (catalog RCB0535). The human AML cell line Kasumi-1, which has *RUNX1:RUNX1T1* and N822K *KIT* mutations, was provided by Hiroshima University. A murine IL-3–dependent myeloid progenitor cell line, 32D, was obtained from the RIKEN cell bank. Human mutant *FLT3*-expressing 32D cells (32D*^mtFLT3^*) were established as previously reported (14). Kasumi-1 and 32D*^mtFLT3^* were used as positive controls for c-KIT and FLT3 expression, respectively (14, 61). All the cell lines were maintained according to the manufacturer’s instructions.

### BMDMs.

Murine bone marrow cells were harvested according to the reported protocol (62) and cultured in Dulbecco’s modified Eagle medium (DMEM) supplemented with 20% FBS, 50 units/mL penicillin, and 50 μg/mL streptomycin. To establish M-CSF–induced BMDMs, 20 ng/mL recombinant mouse M-CSF (PeproTech; catalog 315-02) was added to the culture medium. Similarly, GM-CSF (PeproTech; catalog AF-315-03) was added for GM-CSF–induced BMDM establishment. To obtain M1 macrophages, 100 ng/mL recombinant mouse IFN-γ (R&D Systems, Bio-Techne; catalog 485-MI) and 100 ng/mL LPS (MilliporeSigma; catalog L4391) were applied to GM-CSF–induced BMDMs from day 5 of culture and cultured for an additional 48 hours (63). Similarly, M2 macrophages were generated by stimulating M-CSF–induced BMDMs with 20 ng/mL recombinant mouse IL-4 (BioLegend; catalog 574302) and 20 ng/mL recombinant mouse IL-13 (Miltenyi Biotec; catalog 130-094-639).

### Western blot analysis.

The BMDMs generated with M-CSF on day 7 were rested in DMEM without FBS supplementation for 12 hours. The cells were treated with the indicated concentration of FF-10101 for the last 2 hours and stimulated with 100 ng/mL M-CSF for the last 5 minutes. After being washed with ice-cold PBS(-), the cells were lysed in 2× Laemmli Sample Buffer (Bio-Rad Laboratories; catalog 1610737). BMDMs induced with both M-CSF and GM-CSF and with or without FF-10101 were lysed 1 hour after stimulation with IFN-γ (50 ng/mL) for signaling pathway analysis.

For drug removal experiments, RAW264 cells were cultured in DMEM without FBS for 12 hours as described above. For the drug removal (+) groups, the cells were cultured with DMSO, FF-10101, or BLZ945 for 2 hours and washed with PBS(-) 3 times. After drug removal, the cells were cultured with DMEM for 10 hours. In the drug removal (-) group, the cells were cultured with the drug for the last 2 hours before M-CSF stimulation.

All the cell lysates were separated by SDS-PAGE and transferred onto polyvinylidene difluoride membranes. Proteins were detected by incubation with primary antibodies followed by incubation with a horseradish peroxidase–linked secondary antibody. The list of antibodies used is summarized in Supplemental Table 2. Images were obtained using a LuminoGraph I (ATTO Corporation; catalog WSE-6100H). The quantification of signal bands from Western blotting was performed with ImageJ version 13.0.6 (National Institutes of Health) and its macro plug-in (64).

### Animal models.

Female C57BL/6J and BALB/c mice were purchased from Charles River Laboratories Japan at 6 weeks of age and used at 7 weeks of age. B6.Cg-*Rag2^tm1.1Cgn^*/J mice were obtained from The Jackson Laboratory. The mice were inoculated subcutaneously with a suspension of 1 × 10^6^ tumor cells in 100 μL of PBS(-) at the right flank (day 0). In some groups, 0.1 mg/mL FF-10101 or BLZ945 was administered with a water supplement bottle for free access. Anti–PD-1 mAb (BioLegend; Clone: RMP1-14; 200 μg per mouse) was administered intraperitoneally on days 6 and 9. For T cell depletion, anti-CD4 mAb (BioXCell; Clone: GK1.5; 200 μg per body) and anti-CD8β mAb (BioXCell; Clone: 53–5.8, 200 μg per body) was injected intraperitoneally on days 4, 8, 12, and 16. The tumor size (mm^2^) was calculated as the length × width. The mice were monitored 3 times a week and euthanized when the tumor length reached greater than 20 mm. In some experiments, tumor tissues were collected on day 3, 8, or 10 after tumor inoculation and treated with Horizon Dri Tumor & Tissue Dissociation Reagent (BD Biosciences; catalog 661563) to obtain tumor-infiltrating lymphocytes for FCM assays. At least 2 independent experiments were conducted with at least 3 biological replicates. All the mice were maintained in a specific pathogen–free animal facility at Nagoya University.

### PIA assay.

The PIA assay was performed as previously reported (27). Female C57BL/6J mice at 7 weeks of age were treated with FF-10101 for 5 days, and their blood was collected. The plasma was isolated by centrifugation at 300*g* for 5 minutes. The 32D*^mtFLT3^* cell line, which shows constitutive activation of FLT3, was incubated with the indicated concentration of FF-10101 or the plasma for 2 hours. After being washed with ice-cold PBS(-), the cells were lysed in 2× Laemmli sample buffer and subjected to Western blot analysis.

### Tumor bulk RNA sequences and data processing.

Tumor samples from MCA205 tumor–bearing mice treated with or without FF-10101 were collected into RNAlater Stabilization Solution (Thermo Fisher Scientific; catalog AM7024), and total RNA was extracted using an RNeasy Plus Mini Kit (QIAGEN; catalog 74134). Tumor RNA was sequenced by Veritas Genetics using an Illumina NovaSeq 6000, and data processing was performed using the Galaxy web platform (65) and R version 4.1.1 (R Foundation for Statistical Computing).

Raw FASTQ files were processed using fastp version 0.19.5 (66), and transcript abundance was quantified using Salmon version 1.5.1 (67). Differentially expressed genes were determined by DESeq2 version 1.22.1 (68). GSEA (69) and CYBERSORTx (70) were performed according to the instructions. The annotated GTF files (GENCODE vM27) were obtained from https://www.gencodegenes.org

### Quantitative real-time PCR.

cDNA was prepared from RNA extracted from the TAM (CD45^+^CD11b^+^F4/80^+^) fraction in tumor samples using SuperScript VILO Master Mix (Thermo Fisher Scientific; catalog 11756050). Quantitative real-time PCR was performed with the originally designed primers listed in Supplemental Table 3, THUNDERBIRD SYBER qPCR Mix (TOYOBO; catalog QPS-201), and a ViiA 7 real-time PCR system (Thermo Fisher Scientific). Mouse *Actb* was used as an internal control.

### Staining for the FCM assay.

FCM staining and analyses were performed as previously described (71, 72). The antibodies and dyes used in the FCM analyses are summarized in Supplemental Tables 4 and 5. The cells were washed with PBS supplemented with 2% FBS and subjected to staining with surface antibodies (1:100 dilution). Intracellular staining of Foxp3 was performed with a mAb against Foxp3 (1:100 dilution) and an eBioscience Foxp3/Transcription Factor Staining Buffer Set (Thermo Fisher Scientific; catalog 00-5523-00) according to the manufacturer’s instructions. After washing, the cells were analyzed with a FACSymphony A3 (BD Biosciences) and FlowJo version 10.6.1 (BD Biosciences).

The cells were stimulated for 4 hours with 100 ng/mL PMA (MilliporeSigma; catalog P8139), 1 μg/mL ionomycin (MilliporeSigma; catalog I0634), and 1× brefeldin A solution (BioLegend; catalog 420601). The cells were stained for cell surface markers and subjected to fixation and permeabilization using a Cytofix/Cytoperm Fixation/Permeabilization Solution Kit (BD Biosciences; catalog 554714). After permeabilization, intracellular staining of cytokines was performed.

To examine antigen-specific CD8^+^ T cells, H2-Kb/SIINFEKL-tetramer-PE was employed. The tetramer was prepared using Flex-T Biotin H-2Kb OVA (SIINFEKL) Monomer (BioLegend; catalog 280051) and PE Streptavidin (BioLegend; catalog 405204) according to the manufacturer’s instructions. The cells were stained for cell surface markers after tetramer staining for 30 minutes at 4°C, then analyzed with a FACSymphony A3 (BD Biosciences) and FlowJo version 10.6.1 (BD Biosciences).

### Proliferation assay.

CD8^+^ T cells, CD4^+^CD25^–^ (T_conv_) cells, and CD4^+^CD25^+^ T (Treg) cells were sorted from the splenocytes of C57BL/6J mice using mouse CD8a MicroBeads (Miltenyi Biotec; catalog 130-117-044), CD4 MicroBeads (Miltenyi Biotec; catalog 130-117-043), biotin anti-mouse CD25 mAb (BioLegend; catalog 102003), and anti-biotin MicroBeads (Miltenyi Biotec; catalog 130-090-485), according to the manufacturers’ instructions. After labeling with CytoTell Blue (AAT Bioquest; catalog 22252), 2 × 10^5^ cells/well were cultured in RPMI 1640 medium supplemented with 10% FBS, 2 mM l-glutamine (Thermo Fisher Scientific; catalog 25030-081), 10 μM 2-ME, and Dynabeads Mouse T-Activator CD3/CD28 (Thermo Fisher Scientific; catalog 11452D) at a bead-to-cell ratio of 1:1 in the presence of FF-10101. Recombinant mouse IL-2 was added at 60 U/mL for the culture of CD8^+^ T cells or CD4^+^CD25^–^ T cells and 100 U/mL for the culture of CD4^+^CD25^+^ cells. Forty-eight hours after incubation, the proliferation and viability of the cells were analyzed with a FACSymphony A3 (BD Biosciences) and FlowJo version 10.6.1 (BD Biosciences).

The direct impact of FF-10101 on tumor cell and BMDM proliferation was evaluated using a Cell Proliferation Kit II (XTT) (MilliporeSigma; catalog 11465015001) according to the manufacturer’s instructions. The DMSO concentration was maintained at 0.05% during the serial drug dilution. The absorbance of the XTT-labeled mixture was measured with a MULTISKAN GO (Thermo Fisher Scientific). Since BMDMs, especially after differentiation into M1 or M2 macrophages, are fragile, BMDMs were first transferred to 96-well plates on day 5 of culture and stimulated for an additional 2 days with IFN-γ + LPS or IL-4 + IL-13. Established M1 and M2 macrophages were treated with the indicated concentrations of FF-10101 and cultured for 48 hours before the XTT assay.

### Ex vivo human cell culture.

Primary endometrial cancer tissues were minced and treated with Horizon Dri Tumor & Tissue Dissociation Reagent (BD Biosciences; catalog 661563) and a MojoSort Human Dead Cell Removal Kit (BioLegend; catalog 480159) according to the manufacturer’s instructions. Patient characteristics are shown in Supplemental Table 6. A total of 2 × 10^5^ cells extracted from tumors were suspended in 200 μL of DMEM containing 20% CTS Immune Cell SR (Thermo Fisher Scientific; catalog A2596101) and were treated with or without 10 nM FF-10101 for 48 hours. TAMs (CD45^+^CD3^–^CD11b^+^CD14^+^) were analyzed and sorted with a FACSymphony S6 (BD Biosciences) and FlowJo version 10.6.1 (BD Biosciences). cDNA was prepared from sorted cells using a QuantAccuracy RT-RamDA cDNA Synthesis Kit (TOYOBO; catalog RMQ-101). Quantitative real-time PCR was performed with the designed primers listed in Supplemental Table 3, THUNDERBIRD SYBER qPCR Mix (TOYOBO; catalog QPS-201), and a ViiA 7 real-time PCR system (Thermo Fisher Scientific). Human *GAPDH* was used as an internal control.

### Statistics.

The relationships between groups were compared using unpaired *t* test, except that paired 2-tailed *t* test was used for ex vivo assays of human samples. Two-way ANOVA and Bonferroni’s multiple comparisons test were applied in grouped analyses for tumor growth kinetics. Statistical analyses were performed with GraphPad Prism 9 (GraphPad Software) or R version 4.1.1, and the data are presented as the means ± SDs. *P* values less than 0.05 were considered to indicate statistical significance.

### Study approval.

Human samples were collected according to a protocol approved by the institutional review board of Nagoya University (#2018-0400). All donors provided written informed consent before sampling. This study was conducted in accordance with ethics guidelines, including the Declaration of Helsinki. Animal care and experiments were performed in accordance with the guidelines of the Division of Experimental Animals, Nagoya University. All animal experiments were conducted in accordance with an approved protocol (#20323, #M210444, and #M220278) reviewed by the Institutional Animal Care and Use Committee.

### Data availability.

The raw sequencing data from this study have been deposited in NCBI GEO and are accessible through accession number GSE242937. Values for all plot data shown in graphs can be found in the Supporting Data Values file.

## Author contributions

HK and H Nishikawa conceptualized the study. TS, DS, YK, TK, HK, and H Nishikawa designed the research. TS and DS conducted experiments and acquired data. TS and JK analyzed data. Data curation, validation, and visualization were conducted by TS, DS, JK, and YK. TS, DS, SH, KS, H Nishinakamura, TI, and YI provided resources. Writing of the original draft and preparation were contributed by TS. Review and editing of the manuscript were contributed by TS, DS, JK, YK, SH, KS, H Nishinakamura, TI, YI, TK, HK, and H Nishikawa. TK, HK, and H Nishikawa contributed supervision. H Nishikawa contributed project administration and funding acquisition.

## Supplementary Material

Supplemental data

Unedited blot and gel images

Supporting data values

## Version 1 01/09/2025

Electronic publication
