# Supplementary material for: Sustained inhibition of CSF1R signaling augments antitumor immunity through inhibiting tumor-associated macrophages
Source: JCI Insight. 2025 Jan 9;10(1):e178146. doi: 10.1172/jci.insight.178146 (PMC11721313; doi:10.1172/jci.insight.178146)
Supplement: Supplemental data [file jciinsight-10-178146-s046.pdf]

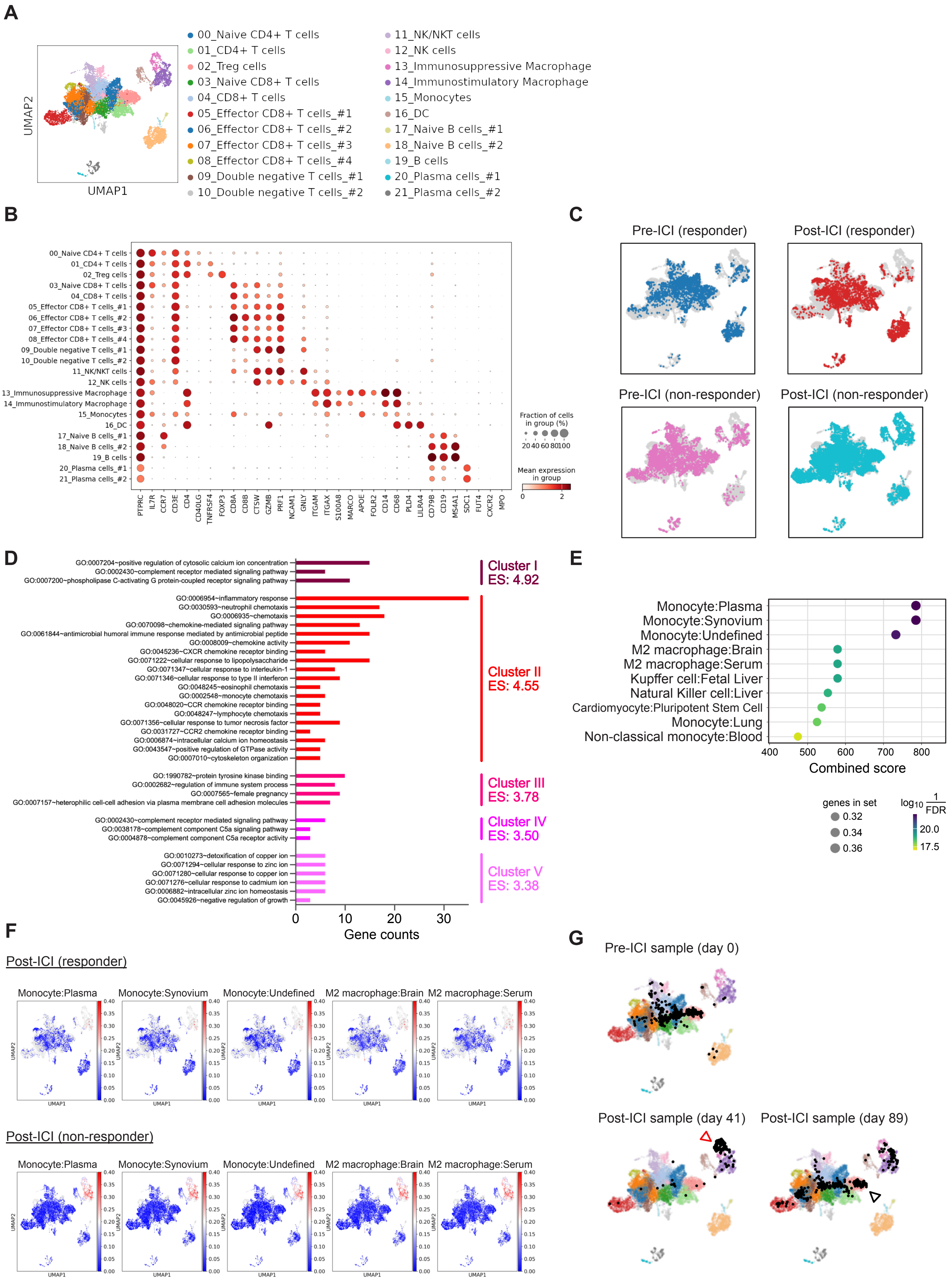
A
UMAP2
UMAP1
00_Naive CD4+ T cells
01_CD4+ T cells
02_Treg cells
03_Naive CD8+ T cells
04_CD8+ T cells
05_Effector CD8+ T cells_#1
06_Effector CD8+ T cells_#2
07_Effector CD8+ T cells_#3
08_Effector CD8+ T cells_#4
09_Double negative T cells_#1
10_Double negative T cells_#2
11_NK/NKT cells
12_NK cells
13_Immunosuppressive Macrophage
14_Immunostimulatory Macrophage
15_Monocytes
16_DC
17_Naive B cells_#1
18_Naive B cells_#2
19_B cells
20_Plasma cells_#1
21_Plasma cells_#2
B
PTPRC IL7R CCR7 CD3E CD4 CD40LG TNFRSF4 FOXP3 CD8A CD8B CTSW GZMB PRF1 NCAM1 GNLY ITGAM ITGAX S100A8 MARCO APOE FOLR2 CD14 CD68 PLD4 LILRA4 CD79B CD19 MS4A1 SDC1 FUT4 CXCR2 MPO
Fraction of cells in group (%)
20 40 60 80 100
Mean expression in group
0 2
C
Pre-ICI (responder)
Post-ICI (responder)
Pre-ICI (non-responder)
Post-ICI (non-responder)
D
GO:0007204~positive regulation of cytosolic calcium ion concentration
GO:0002430~complement receptor mediated signaling pathway
GO:0007200~phospholipase C-activating G protein-coupled receptor signaling pathway
Cluster I
ES: 4.92
GO:0006954~inflammatory response
GO:0030593~neutrophil chemotaxis
GO:0006935~chemotaxis
GO:0070098~chemokine-mediated signaling pathway
GO:0061844~antimicrobial humoral immune response mediated by antimicrobial peptide
GO:0008009~chemokine activity
GO:0045236~CXCR chemokine receptor binding
GO:0071222~cellular response to lipopolysaccharide
GO:0071347~cellular response to interleukin-1
GO:0071346~cellular response to type II interferon
GO:0048245~eosinophil chemotaxis
GO:0002548~monocyte chemotaxis
GO:0048020~CCR chemokine receptor binding
GO:0048247~lymphocyte chemotaxis
GO:0071356~cellular response to tumor necrosis factor
GO:0031727~CCR2 chemokine receptor binding
GO:0006874~intracellular calcium ion homeostasis
GO:0043547~positive regulation of GTPase activity
GO:0007010~cytoskeleton organization
Cluster II
ES: 4.55
GO:1990782~protein tyrosine kinase binding
GO:0002682~regulation of immune system process
GO:0007565~female pregnancy
GO:0007157~heterophilic cell-cell adhesion via plasma membrane cell adhesion molecules
Cluster III
ES: 3.78
GO:0002430~complement receptor mediated signaling pathway
GO:0038178~complement component C5a signaling pathway
GO:0004878~complement component C5a receptor activity
Cluster IV
ES: 3.50
GO:0010273~detoxification of copper ion
GO:0071294~cellular response to zinc ion
GO:0071280~cellular response to copper ion
GO:0071276~cellular response to cadmium ion
GO:0006882~intracellular zinc ion homeostasis
GO:0045926~negative regulation of growth
Cluster V
ES: 3.38
0 10 20 30
Gene counts
E
Monocyte:Plasma
Monocyte:Synovium
Monocyte:Undefined
M2 macrophage:Brain
M2 macrophage:Serum
Kupffer cell:Fetal Liver
Natural Killer cell:Liver
Cardiomyocyte:Pluripotent Stem Cell
Monocyte:Lung
Non-classical monocyte:Blood
400 500 600 700 800
Combined score
genes in set
0.32
0.34
0.36
$\log_{10}\frac{1}{FDR}$
20.0
17.5
F
Post-ICI (responder)
Monocyte:Plasma
Monocyte:Synovium
Monocyte:Undefined
M2 macrophage:Brain
M2 macrophage:Serum
Post-ICI (non-responder)
Monocyte:Plasma
Monocyte:Synovium
Monocyte:Undefined
M2 macrophage:Brain
M2 macrophage:Serum
0.40 0.35 0.30 0.25 0.20 0.15 0.10 0.05 0.00
UMAP1
UMAP2
G
Pre-ICI sample (day 0)
Post-ICI sample (day 41)
Post-ICI sample (day 89)

H

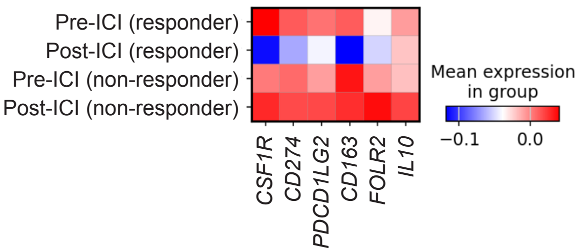

I

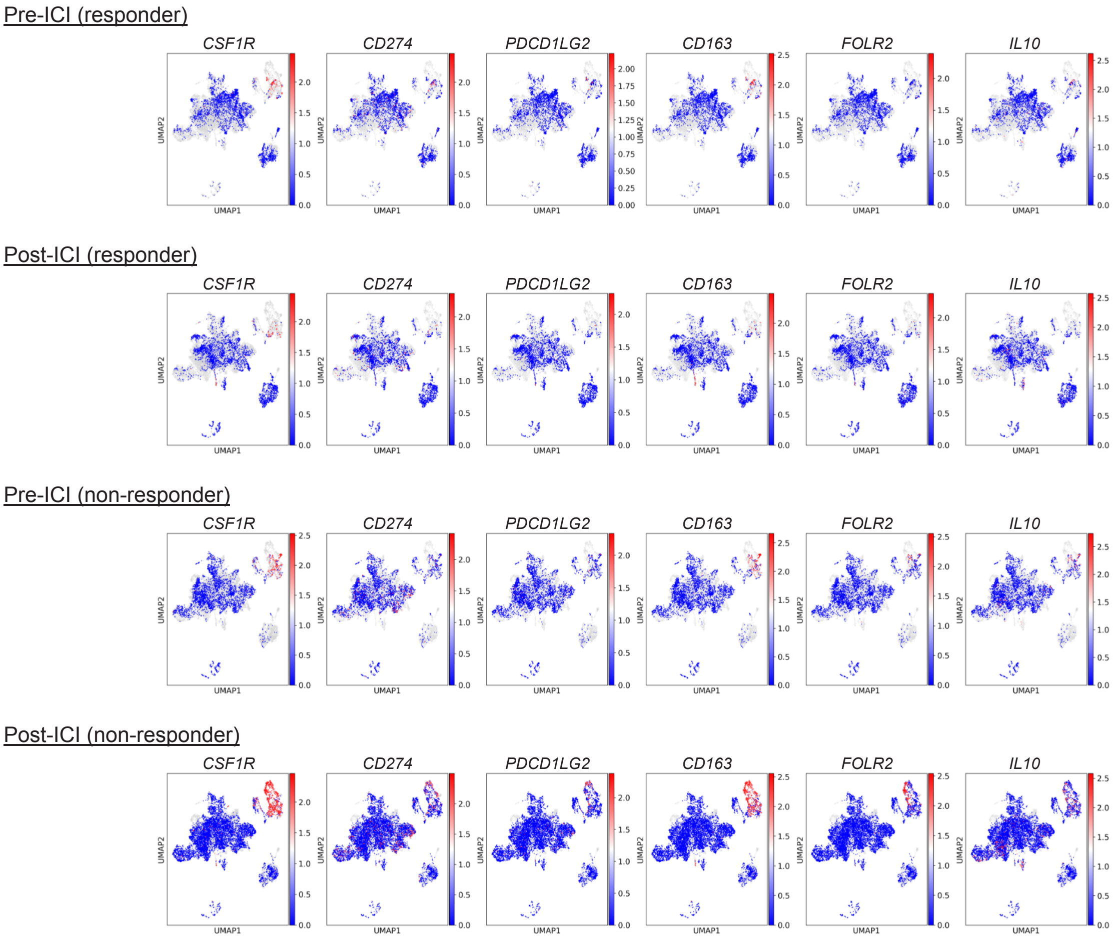

Supplemental Figure 1. Non-responders to ICIs harbor high expression of immunosuppressive macrophage marker genes in CSF1R$^+$ clusters.
(A) UMAP visualization of clusters identified by integrating the transcriptomes of tumor samples obtained before and after ICI treatment. Twenty-two clusters, which were defined by the Leiden algorithm, are shown in different colors. **(B)** Dot plot depicting the percentage of cells expressing the indicated molecules and the mean expression of representative genes in each cluster. **(C)** UMAP visualization based on clinical annotations according to sampling points and clinical response to ICIs. **(D)** Genes whose expression was significantly increased in non-responders were explored via DAVID analysis. The top 5 clusters are shown with their enrichment scores (ES). The bar plot represents the gene counts of each gene set with an adjusted *P* value less than 0.05. **(E)** GSEA with the “CellMarker_Augmented_2021” gene set library from *Enrichr* was performed to compare post-ICI treatment samples of non-responders with those of responders. The top 10 gene sets significantly enriched in non-responders are shown as dot plots with combined scores. **(F)** Intensities of the top 5 enriched gene sets visualized over the UMAP plot. High-intensity scores are shown in red. **(G)** Representative sequential analyses of non-responder. Black highlighted dots represent the cells derived from Case #28, who was ICI resistant. Immunosuppressive TAMs (red arrowhead) are induced in the early phase of ICI tolerance, followed by Treg accumulation (black arrowhead). **(H)** Heatmap analysis of immunosuppressive macrophage-related genes. **(I)** UMAP visualization of immunosuppressive macrophage-related genes and *CSF1R* on the basis of clinical annotations according to sampling points and clinical response to ICIs.

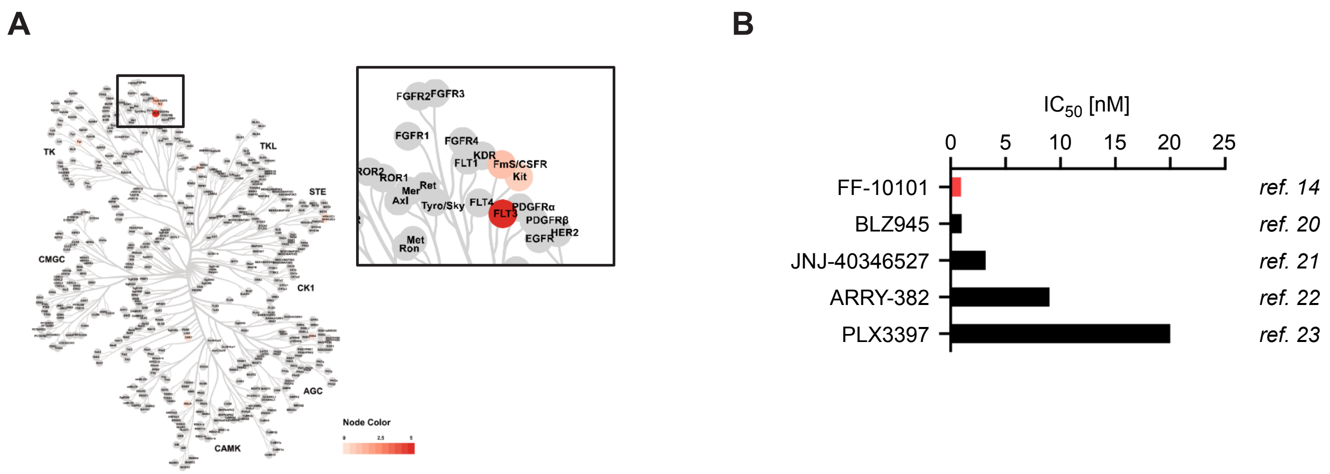

Supplemental Figure 2. FF-10101 is a kinase inhibitor with high selectivity and potent inhibitory activity.

(A) Kinome map of FF-10101. An enlarged view of the square area, including FLT3, CSF1R, and KIT, is shown on the right side. **(B)** Comparison of the IC_{50} values against CSF1R among various CSF1R inhibitors. The reference articles are shown on the right side.

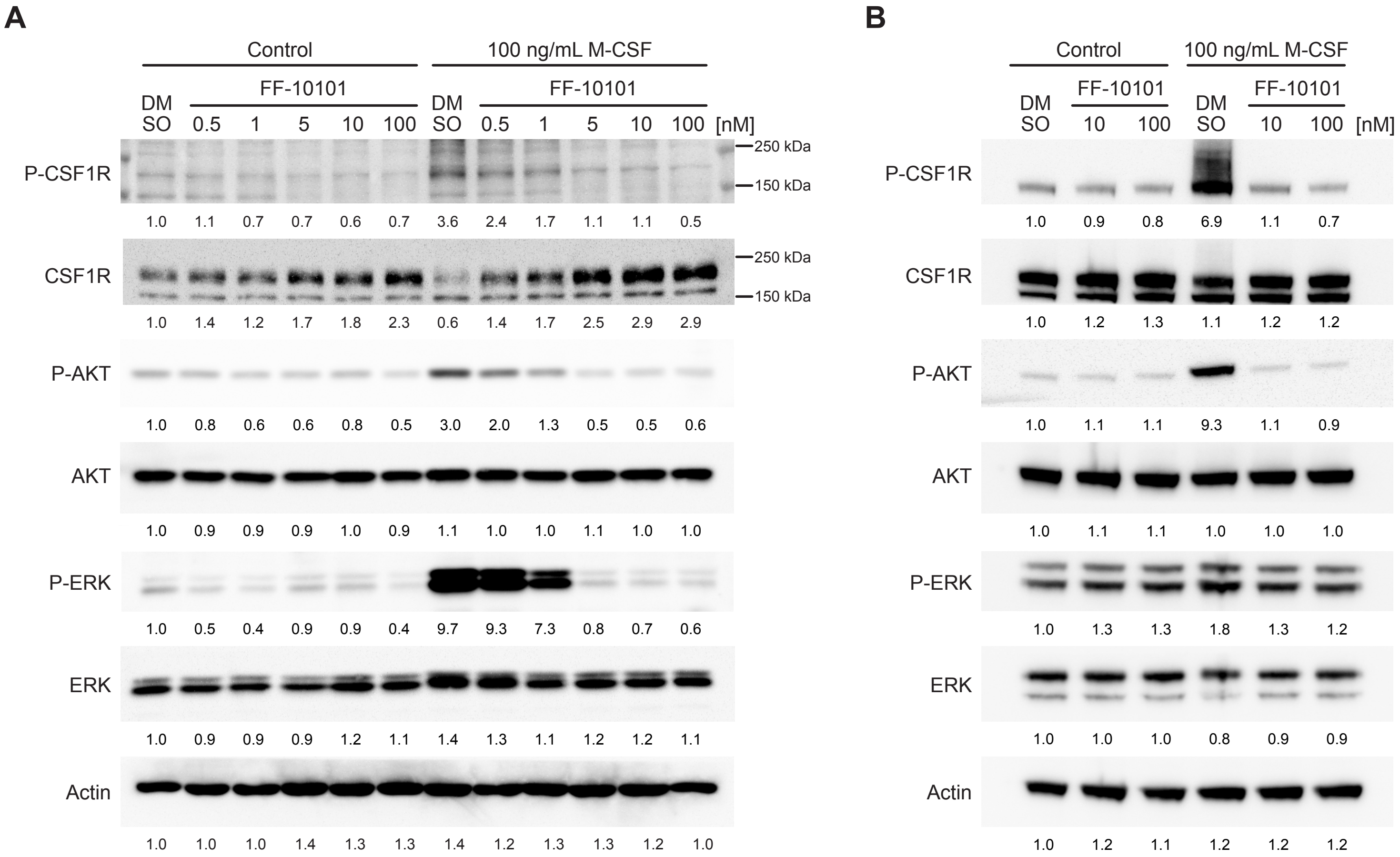

Supplemental Figure 3. FF-10101 harbors a strong inhibitory activity against CSF1R.
Western blot analyses showing the inhibition of CSF1R by FF-10101 in human monocyte-derived macrophages (**A**) and the RAW264 cell line (**B**). Human monocytes were isolated from peripheral blood mononuclear cells using anti-CD14 MicroBeads (Miltenyi Biotec; Cat. #130-050-201) and cultured in RPMI 1640 medium supplemented with 10% FBS and 20 ng/mL recombinant human M-CSF. The human monocyte-derived macrophages were rested in RPMI 1640 without FBS supplementation for 12 hours. The cells were treated with the indicated concentration of FF-10101 for the last 2 hours and stimulated with 100 ng/mL recombinant M-CSF for the last 5 minutes. After being washed with ice-cold PBS(-), the cells were lysed in 2× Laemmli Sample Buffer (Bio-Rad Laboratories; Cat. #1610737) for Western blot analyses.

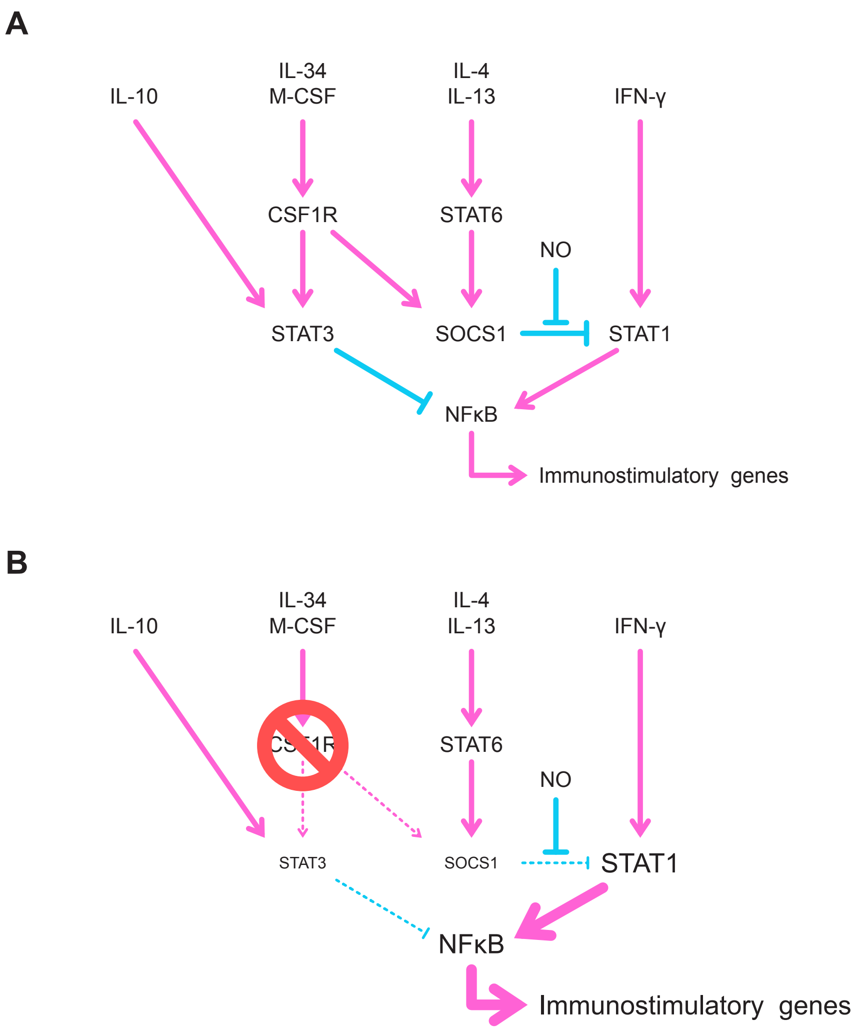

Supplemental Figure 4. FF-10101 treatment induces changes in signaling pathways.
(A) Downstream signaling of CSF1R and related cytokines reported to be involved in TAM polarization.
(B) Signaling changes induced by FF-10101.

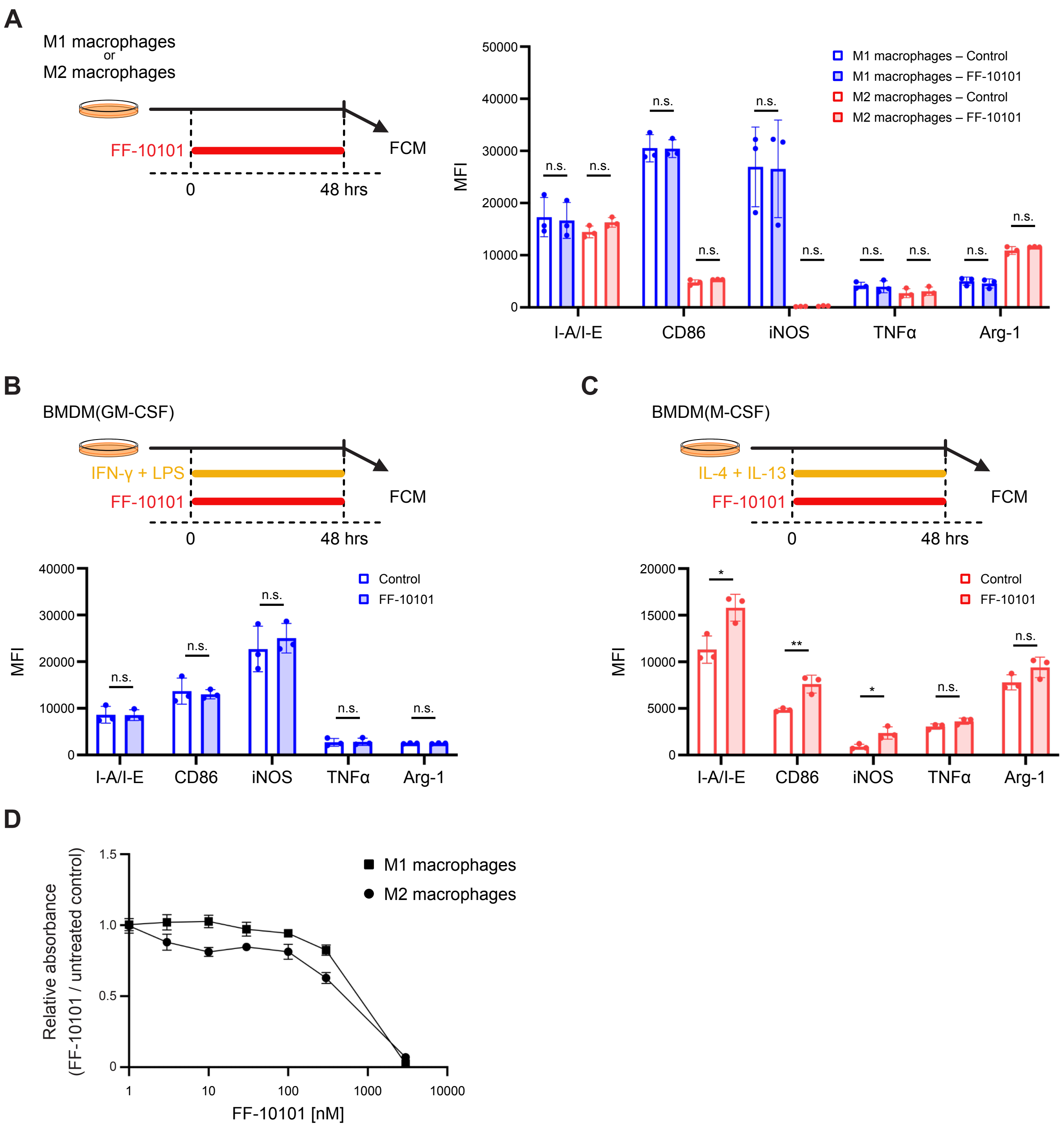

Supplemental Figure 5. FF-10101 affects BMDM polarization but has a limited effect on established M1 or M2 macrophages.

(A) Experimental scheme (left) and expression of cell surface molecules on pre-estalished M1 and M2 macrophages (right). The macrophages were treated with FF-10101 for 48 hours. **(B and C)** Experimental scheme (top) and expression of cell surface molecules (bottom). FF-10101 was applied at the onset of M1 (**B**) and M2 (**C**) macrophage induction. The bar plots are shown as the means ± SDs and were compared by unpaired *t* tests. *P* values: n.s. ≥ 0.05, * < 0.05, ** < 0.01. MFI, mean fluorescence intensity. **(D)** The viability of pre-established M1 and M2 macrophages cultured with the indicated concentrations of FF-10101 was evaluated with an XTT assay. The relative absorbance (FF-10101/ untreated control) of the XTT-labeled mixture is shown.

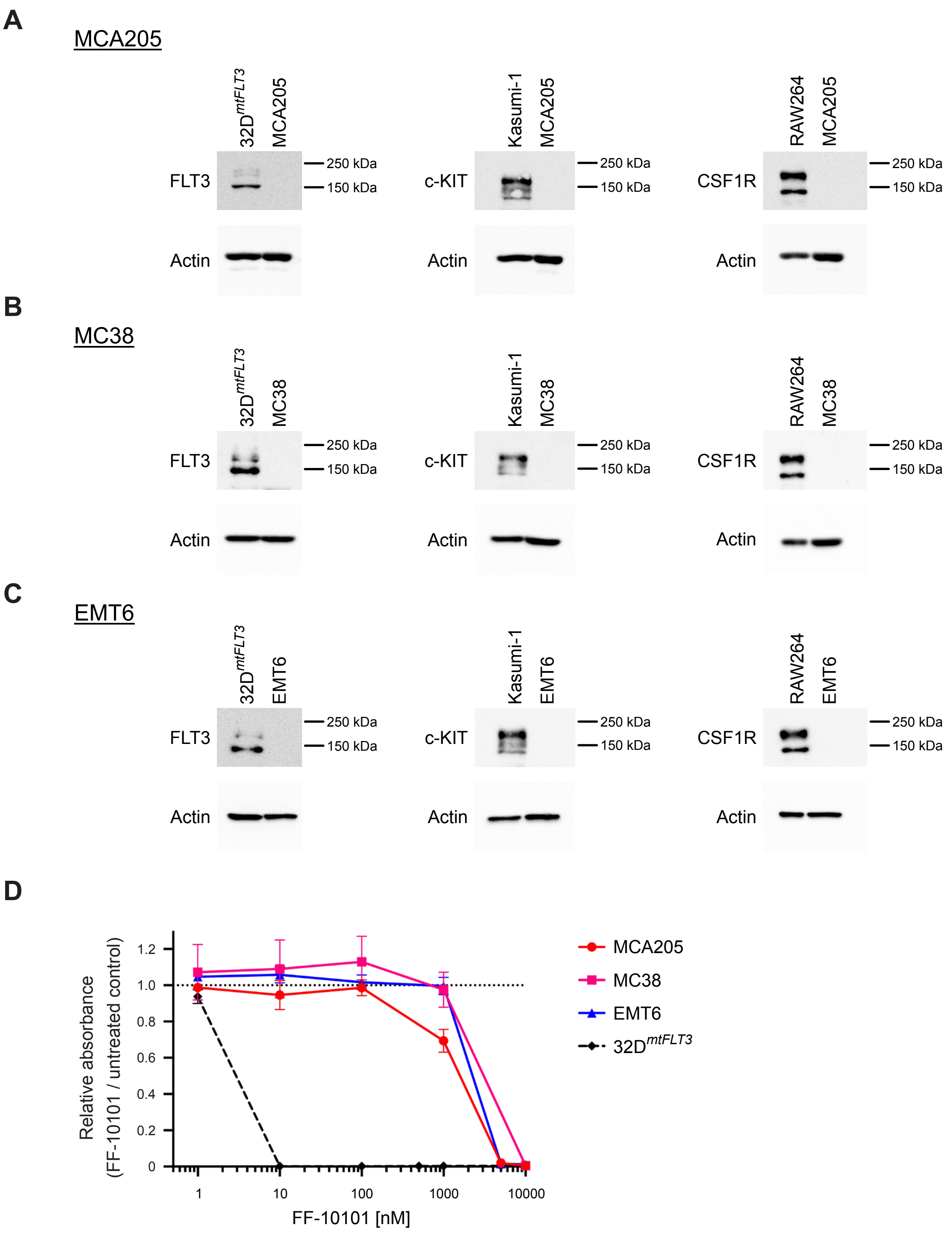

Supplemental Figure 6. FF-10101 has no direct effect on tumor cell lines.
(A–C) Western blot analyses showing the expression of FLT3, c-KIT, and CSF1R by the MCA205 (**A**), MC38 (**B**), and EMT6 (**C**) cell lines. $32D^{mtFLT3}$, Kasumi-1, and RAW264 cells were used as positive controls. **(D)** The viability of tumor cells cultured with the indicated concentrations of FF-10101 was evaluated with an XTT assay. The relative absorbance (FF-10101/untreated control) of the XTT-labeled mixture is shown. $32D^{mtFLT3}$ was used as a representative cell line that was sensitive to FF-10101.

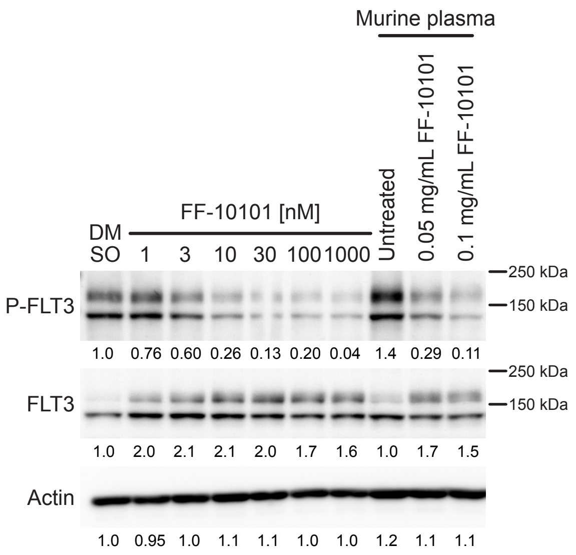

Supplemental Figure 7. FF-10101 is properly absorbed by mice via the free-drinking water.

Plasma harvested from FF-10101-treated mice was isolated and applied to the constitutively FLT3-activated cell line 32D^{mtFLT3}. The cells were also treated with the indicated concentrations of FF-10101 to estimate the blood concentration.

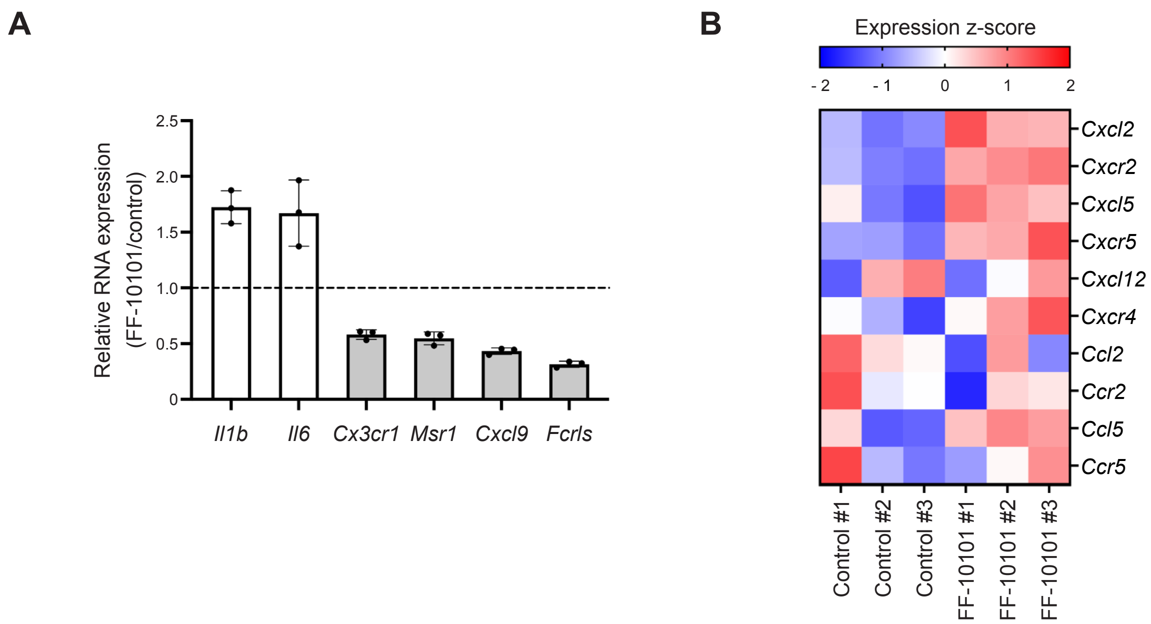

Supplemental Figure 8. The expression of M1-like macrophage-related genes increased after FF-10101 treatment.

(A) RNA expression of the indicated molecules was examined using quantitative real-time PCR. The means of relative RNA expression are shown as FF-10101/control ± SDs. **(B)** Heatmap of representative myeloid chemokine genes.

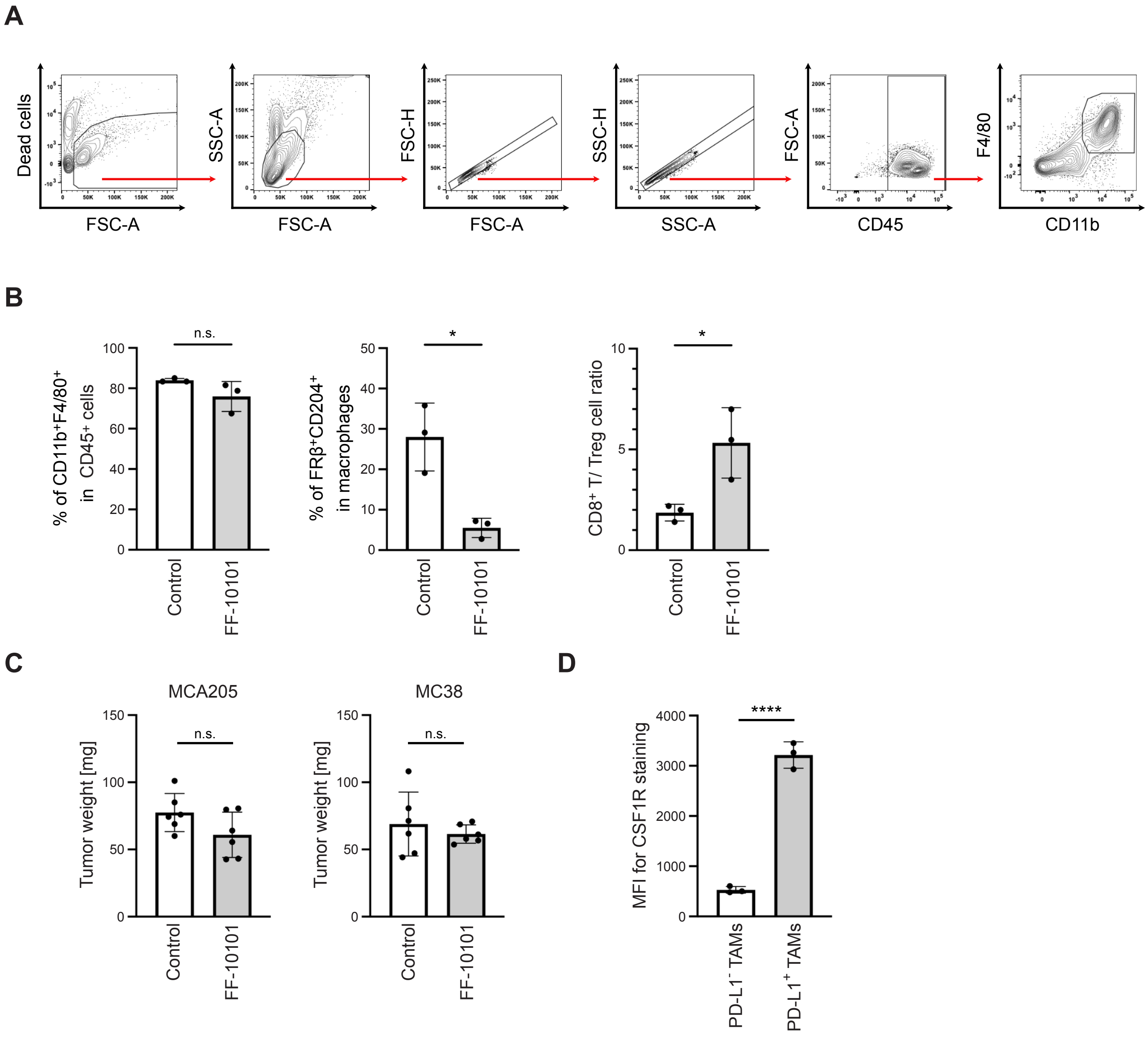

Supplemental Figure 9. Immunosuppressive TAMs are reduced by FF-10101 treatment. **(A)** Gating strategy for the $CD45^+CD11b^+F4/80^+$ fraction (TAMs) used in this study. **(B)** FCM analyses of immune cells collected from tumors on day 3. **(C)** Tumor weights measured on day 8 after tumor inoculation. **(D)** The expression of CSF1R on PD-L1$^+$ and PD-L1$^-$ TAMs. The bar plots are shown as the means ± SDs and were compared by unpaired *t* tests. *P* values: n.s. ≥ 0.05, * < 0.05, **** < 0.0001. MFI, mean fluorescence intensity.

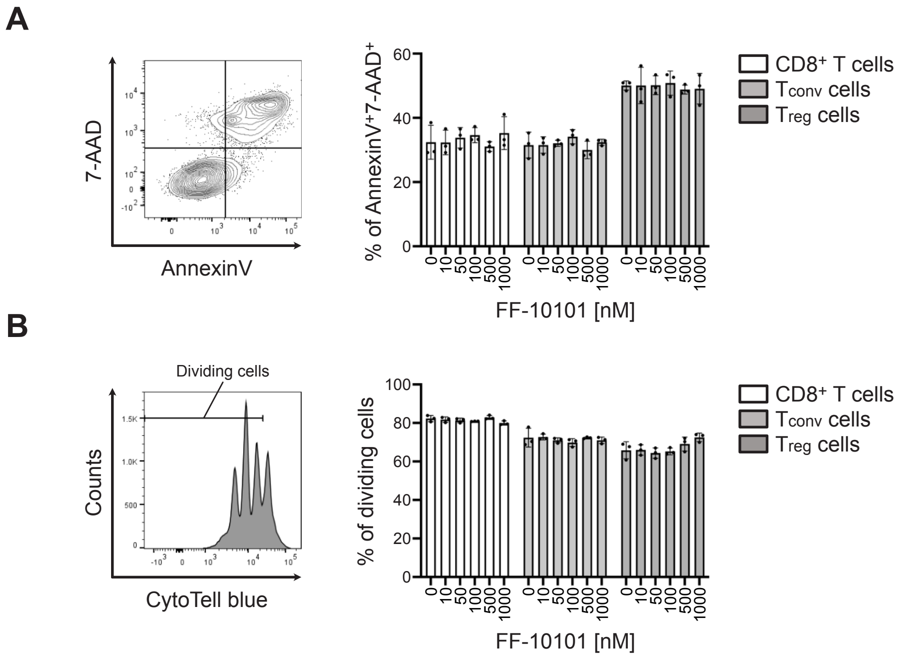

Supplemental Figure 10. FF-10101 shows no direct effect on T cell populations.
(A and **B)** Viability (**A**) and cell proliferation (**B**) of each T cell fraction. CD8$^+$ T cells, conventional CD4$^+$ T (T_{conv}: CD4$^+$CD25$^-$) cells, and Treg (CD4$^+$CD25$^+$) cells were sorted and cultured for 48 hours with the indicated concentrations of FF-10101. A representative contour plot or histogram (left) and summaries (right) are shown.

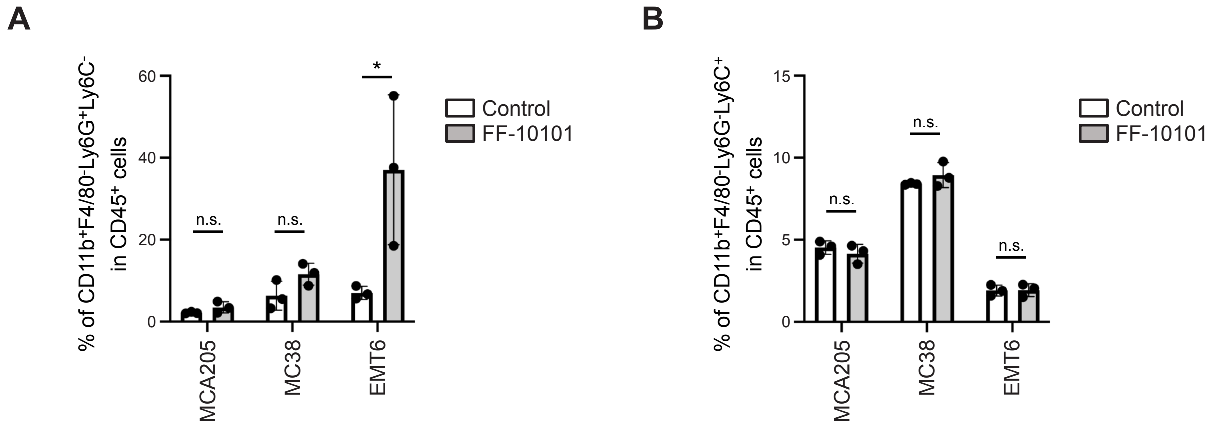

Supplemental Figure 11. The compensatory recruitment of PMN-MDSCs varies among cell lines.

(A and **B)** Changes in PMN-MDSC ($CD45^{+}CD11b^{+}F4/80^{-}Ly6G^{+}Ly6C^{-}$ cell) population (**A**) and M-MDSC ($CD45^{+}CD11b^{+}F4/80^{-}Ly6G^{-}Ly6C^{+}$ cell) population **(B)** in the TME induced by FF-10101 treatment in the indicated tumor models. The bar plots are shown as the means ± SDs and were compared by unpaired *t* tests. *P* values: n.s. ≥ 0.05, * < 0.05.

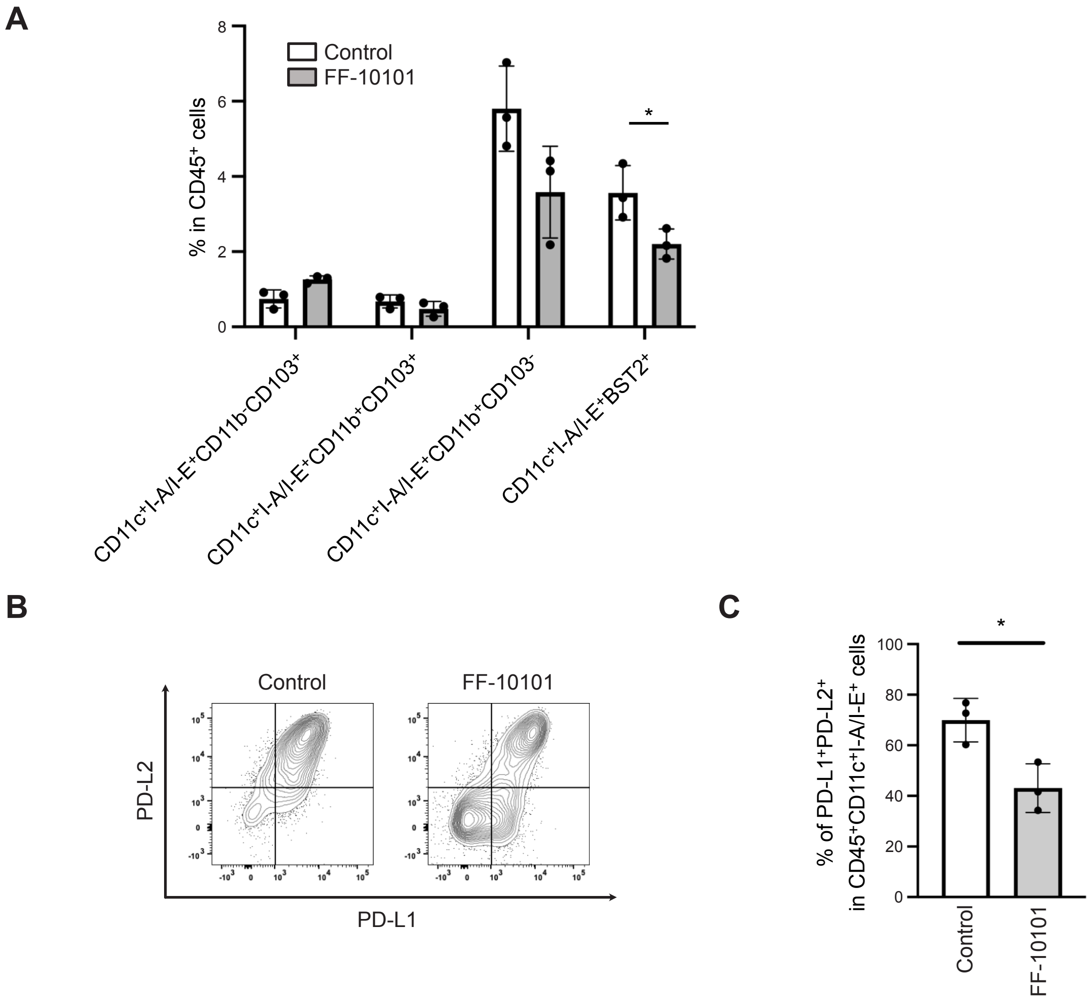

Supplemental Figure 12. FF-10101 treatment reduces a specific type of dendritic cells (DCs).

(A) Summaries of the frequencies of CD11c^{+}I-A/I-E^{+}CD11b^{-}CD103^{+} cells, CD11c^{+}I-A/I-E^{+}CD11b^{+}CD103^{+} cells, CD11c^{+}I-A/I-E^{+}CD11b^{+}CD103^{-} cells, and CD11c^{+}I-A/I-E^{+}BST2^{+} cells in CD45^{+} cells with or without FF-10101 treatment. The experimental scheme is shown in **Figure 5A**. **(B)** Representative contour plots of PD-L1 and PD-L2 expression on CD45^{+}CD11c^{+}I-A/I-E^{+} cells (DCs). **(C)** Summaries of the frequency of PD-L1^{+}PD-L2^{+} cells in DCs with or without FF-10101 treatment. Statistical analyses were performed by unpaired *t* test; * $P < 0.05$.

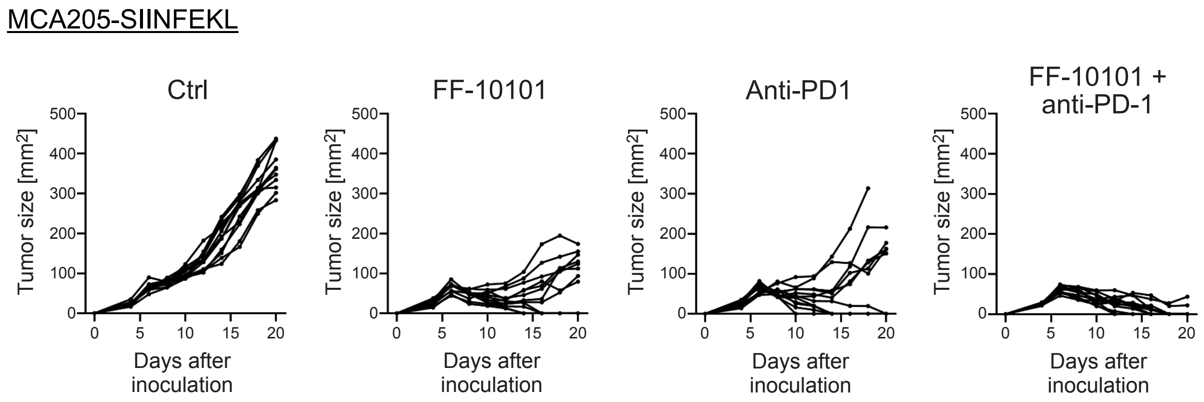

Supplemental Figure 13. Combination treatment with FF-10101 and anti-PD-1 mAb exhibits a strong antitumor effect.

The tumor growth curves for each mouse shown in **Figure 8B**. One million MCA205-SIINFEKL cells were inoculated into the mice on day 0. Some mice received FF-10101 treatment and/or anti-PD-1 mAb treatment.

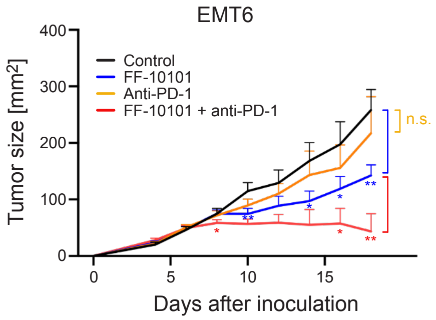

Supplemental Figure 14. Combination treatment with FF-10101 and anti-PD-1 mAb exhibits a strong antitumor effect in EMT6 model.
Tumor growth curves for mice inoculated with EMT6 cells. The mice were inoculated with one million EMT6 cells, and some of the mice received FF-10101 treatment and/or anti-PD-1 mAb treatment. Comparisons between two groups were conducted by two-way ANOVA with multiple *t* tests corrected with Bonferroni's method. Adjusted *P* values: n.s. ≥ 0.05, * < 0.05, ** < 0.01.

Supplemental Tables 1–6

Supplemental Table 1. Immune cell annotation based on known markers.
Positive markers are required for each clustering. Negative markers should be absent or low.

Cluster name	Positive markers	Negative markers
00_Naive CD4+ T cells	*PTPRC, CD3E, CD4, CCR7*	*FOXP3, CD8A, CTSW, NCAM1, ITGAM, CD79B, MPO*
01_ CD4+ T cells	*PTPRC, CD3E, CD4*	*CCR7, FOXP3, CD8A, CTSW, NCAM1, ITGAM, CD79B, MPO*
02_Treg cells	*PTPRC, CD3E, CD4, FOXP3*	*CD8A, CTSW, NCAM1, ITGAM, CD79B, MPO*
03_Naive CD8+ T cells	*PTPRC, CD3E, CD8A, CTSW, CCR7*	*CD4, CD40LG, NCAM1, ITGAM, CD79B, MPO*
04_CD8+ T cells	*PTPRC, CD3E, CD8A, CTSW*	*CCR7, CD4, CD40LG, NCAM1, ITGAM, CD79B, MPO*
05_Effector CD8+ T cells_#1	*PTPRC, CD3E, CD8A, CTSW, GZMB, PRF1*	*CCR7, CD4, CD40LG, NCAM1, ITGAM, CD79B, MPO*
06_ Effector CD8+ T cells_#2	*PTPRC, CD3E, CD8A, CTSW, GZMB, PRF1*	*CCR7, CD4, CD40LG, NCAM1, ITGAM, CD79B, MPO*
07_ Effector CD8+ T cells_#3	*PTPRC, CD3E, CD8A, CTSW, GZMB, PRF1*	*CCR7, CD4, CD40LG, NCAM1, ITGAM, CD79B, MPO*
08_ Effector CD8+ T cells_#4	*PTPRC, CD3E, CD8A, CTSW, GZMB, PRF1*	*CCR7, CD4, CD40LG, NCAM1, ITGAM, CD79B, MPO*
09_Double negative T cells_#1	*PTPRC, CD3E*	*CD4, CD40LG, CD8A, NCAM1, ITGAM, CD79B, MPO*
10_Double negative T cells_#2	*PTPRC, CD3E*	*CD4, CD40LG, CD8A, NCAM1, ITGAM, CD79B, MPO*
11_NK/NKT cells	*PTPRC, NCAM1, GNLY, CD3E, CD4 or CD8A*	*FOXP3, ITGAM, CD79B, MPO*
12_NK cells	*PTPRC, NCAM1, GNLY*	*CD4, CD8A, FOXP3, ITGAM, CD79B, MPO*
13_Immunosuppressive Macrophage	*PTPRC, ITGAM, MARCO, APOE, FOLR2*	*CD3E, NCAM1, CD79B, MPO*
14_Immunostimulatory Macrophage	*PTPRC, ITGAM, MARCO*	*APOE, FOLR2, CD3E, NCAM1, CD79B, MPO*
15_Monocytes	*PTPRC, CD14, CD68*	*MARCO, NCAM1, CD79B, MPO*
16_DC	*PTPRC, PLD4, LILRA4*	*NCAM1, ITGAM, CD79B, MPO*
17_Naive B cells_#1	*PTPRC, CD79B, CD19, MS4A1,*	*CD3E, NCAM1, ITGAM, SDC1,*

	CCR7	*MPO*
18_Naive B cells_#2	*PTPRC, CD79B, CD19, MS4A1, CCR7*	*CD3E, NCAM1, ITGAM, SDC1, MPO*
19_B cells	*PTPRC, CD79B, CD19, MS4A1*	*CCR7, CD3E, NCAM1, ITGAM, SDC1, MPO*
20_Plasma cells_#1	*PTPRC, CD79B, CD19, SDC1*	*CD3E, NCAM1, ITGAM, MPO*
21_Plasma cells_#2	*PTPRC, CD79B, CD19, SDC1*	*CD3E, NCAM1, ITGAM, MPO*

Supplemental Table 2. List of antibodies used for Western blot analyses.

**Antigen**	**Source / Isotype**	**Cat. #**	**Vender**
Phospho-CSF1R (Tyr723)	Rabbit monoclonal IgG	3155	Cell Signaling Technology
CSF1R	Rabbit polyclonal Abs	3152	Cell Signaling Technology
Phospho-FLT3 (Tyr591)	Rabbit polyclonal Abs	3461	Cell Signaling Technology
FLT3	Rabbit monoclonal IgG	3462	Cell Signaling Technology
Phospho-c-KIT (Tyr719)	Rabbit polyclonal Abs	3391	Cell Signaling Technology
c-KIT	Rabbit monoclonal IgG	3074	Cell Signaling Technology
Phospho-AKT (Ser473)	Rabbit polyclonal Abs	9271	Cell Signaling Technology
AKT (pan)	Rabbit monoclonal IgG	4691	Cell Signaling Technology
Phospho-p44/42 MAPK (ERK1/2) (Thr202/Tyr204)	Rabbit polyclonal Abs	9101	Cell Signaling Technology
p44/42 MAPK (ERK1/2)	Rabbit polyclonal Abs	9102	Cell Signaling Technology
SOCS1	Rabbit polyclonal Abs	3950	Cell Signaling Technology
Phospho-STAT1 (Tyr701)	Rabbit monoclonal IgG	7649	Cell Signaling Technology
STAT1	Rabbit monoclonal IgG	14994	Cell Signaling Technology
Phospho-STAT3 (Tyr705)	Rabbit monoclonal IgG	9145	Cell Signaling Technology
STAT3	Rabbit monoclonal IgG	4904	Cell Signaling Technology
Phospho-NF-κB (Ser536)	Rabbit monoclonal IgG	3033	Cell Signaling Technology
NF-κB	Rabbit monoclonal IgG	8242	Cell Signaling Technology
β-Actin	Rabbit monoclonal IgG	4970	Cell Signaling Technology
Anti-rabbit IgG, HRP-linked	Goat	7074	Cell Signaling Technology

Supplemental Table 3. List of primers used in quantitative real-time PCR analyses.

**Gene**	**Organism**	**Direction**	**Sequence (5'–3')**
Il1b	*Mus musculus*	Forward	ACGGACCCCAAAAGATGAAG
Il1b	*Mus musculus*	Reverse	TTCTCCACAGCCACAATGAG
Il6	*Mus musculus*	Forward	GATGCTACCAAACTGGATATAATCAG
Il6	*Mus musculus*	Reverse	CTCTGAAGGACTCTGGCTTTG
Cx3cr1	*Mus musculus*	Forward	GTTATTTGGGCGACATTGTGG
Cx3cr1	*Mus musculus*	Reverse	ATGTCAGTGATGCTCTTGGG
Msr1	*Mus musculus*	Forward	AGTCCGTGAATCTACAGCAAAG
Msr1	*Mus musculus*	Reverse	TCTCACTTCCTGTTTTACTTCCTG
Cxcl9	*Mus musculus*	Forward	AGTCCGCTGTTCTTTTCCTC
Cxcl9	*Mus musculus*	Reverse	TGAGGTCTTTGAGGGATTTGTAG
Fcrls	*Mus musculus*	Forward	CTGTCTCAAAGGGAGCTACTTG
Fcrls	*Mus musculus*	Reverse	TCATAAGAACGGTGTGGAAGG
Actb	*Mus musculus*	Forward	AGTGTGACGTTGACATCCGT
Actb	*Mus musculus*	Reverse	GCAGCTCAGTAACAGTCCGC
IL1B	*Homo sapiens*	Forward	ATGCACCTGTACGATCACTG
IL1B	*Homo sapiens*	Reverse	ACAAAGGACATGGAGAACACC
IL6	*Homo sapiens*	Forward	CCACTCACCTCTTCAGAACG
IL6	*Homo sapiens*	Reverse	CATCTTTGGAAGGTTCAGGTTG
GAPDH	*Homo sapiens*	Forward	CTGACTTCAACAGCGACACC
GAPDH	*Homo sapiens*	Reverse	GTGGTCCAGGGGTCTTACTC

Supplemental Table 4. List of antibodies used in flow cytometry analyses.

Antibody	Fluorophore	Cat. #	Vender
Anti-human CD11b	BUV737	741826	BD Biosciences
Anti-human CD11c	BV786	740966	BD Biosciences
Anti-human CD14	APC-Cy7	561709	BD Biosciences
Anti-human CD274	BUV805	742059	BD Biosciences
Anti-human CD3	Alexa Fluor 700	300423	BioLegend
Anti-human CD45	PerCP-Cy5.5	368503	BioLegend
Anti-mouse Arginase-1	BUV805	368-3697-80	Thermo Fisher Scientific
Anti-mouse CD103	BV650	748256	BD Biosciences
Anti-mouse CD11b	BV711	563168	BD Biosciences
Anti-mouse CD11b	PE-Cy7	101216	BioLegend
Anti-mouse CD11b	Alexa Fluor 700	101222	BioLegend
Anti-mouse CD11c	PE-Cy7	117318	BioLegend
Anti-mouse CD11c	APC-F750	117351	BioLegend
Anti-mouse CD115	PE	135505	BioLegend
Anti-mouse CD204	PE-Dazzle594	154717	BioLegend
Anti-mouse CD223	PE	125207	BioLegend
Anti-mouse CD25	BV605	563061	BD Biosciences
Anti-mouse CD25	BV650	102037	BioLegend
Anti-mouse CD273	BV421	564245	BD Biosciences
Anti-mouse CD274	APC	564715	BD Biosciences
Anti-mouse CD274	PE-CF594	567032	BD Biosciences
Anti-mouse CD3	BUV496	741117	BD Biosciences
Anti-mouse CD3	Alexa Fluor 700	557984	BD Biosciences
Anti-mouse CD3	PerCP-Cy5.5	100217	BioLegend
Anti-mouse CD317	BV421	127023	BioLegend
Anti-mouse CD4	APC-F750	116020	BioLegend
Anti-mouse CD4	BV510	100553	BioLegend
Anti-mouse CD45	BUV496	749889	BD Biosciences
Anti-mouse CD45	FITC	553080	BD Biosciences
Anti-mouse CD45.2	APC	561875	BD Biosciences
Anti-mouse CD8a	BV786	563332	BD Biosciences
Anti-mouse CD86	BV421	564198	BD Biosciences
Anti-mouse F4/80	APC	17-4801-80	Thermo Fisher Scientific
Anti-mouse F4/80	BV785	123141	BioLegend
Anti-mouse FOXP3	PerCP-Cy5.5	45-5773-82	Thermo Fisher Scientific
Anti-mouse FOXP3	PE	12-5773-82	Thermo Fisher Scientific

Anti-mouse FRβ	PE	153303	BioLegend
Anti-mouse I-A/I-E	BV650	107641	BioLegend
Anti-mouse I-A/I-E	AF488	562352	BD Biosciences
Anti-mouse I-A/I-E	PerCP-Cy5.5	107626	BioLegend
Anti-mouse IFN-γ	eFluor450	43-7311-82	Thermo Fisher Scientific
Anti-mouse IL-2	PE-Cy7	25-7021-82	Thermo Fisher Scientific
Anti-mouse iNOS	AF488	53-5920-80	Thermo Fisher Scientific
Anti-mouse iNOS	PE	12-5920-80	Thermo Fisher Scientific
Anti-mouse Ly-6C	BV421	128031	BioLegend
Anti-mouse Ly-6G	BV785	127645	BioLegend
Anti-mouse TNFα	APC	17-7321-82	Thermo Fisher Scientific

Supplemental Table 5. List of protein and dyes used in flow cytometry analyses.

Name	Cat. #	Vender
7-AAD	420403	BioLegend
Annexin V-APC	640919	BioLegend
eBioscience™ Fixable Viability Dye eFluor™ 506	65-0866-14	Thermo Fisher Scientific

Supplemental Table 6. Patient characteristics from whom primary cancer tissues were examined.

No.	Age	Histological type	FIGO* stage	TNM classification	Neoadjuvant therapy
1	52	Endometrioid G1–2	1A	pT1aNxM0	No
2	51	Endometrioid G1–2	1A	pT1aN0M0	No
3	52	Endometrioid G3	3A	pT3aN0M0	No
4	53	Endometrioid G1–2	1B	pT1bN0M0	No
5	58	Endometrioid G1–2	1A	pT1aN0M0	No
6	57	Endometrioid G1–2	1B	pT1bN0M0	No
7	42	Endometrioid G1–2	1A	pT1aN0M0	No
8	68	Endometrioid G3	3A	pT3aN0M0	No
9	64	Carcinosarcoma	1A	pT1aN0M0	No

* FIGO: International Federation of Gynecologists and Obstetricians
