# Supplementary material for: Sustained inhibition of CSF1R signaling augments antitumor immunity through inhibiting tumor-associated macrophages
Source: JCI Insight. 2025 Jan 9;10(1):e178146. doi: 10.1172/jci.insight.178146 (PMC11721313; doi:10.1172/jci.insight.178146)

Full unedited blots for Figure 2A

P-CSF1R

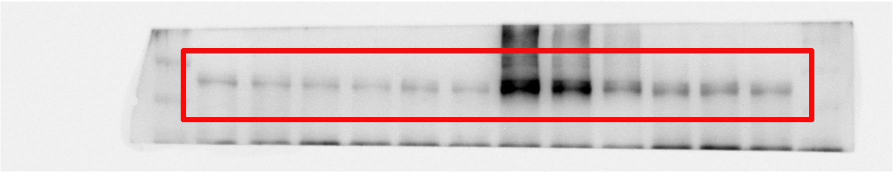

CSF1R

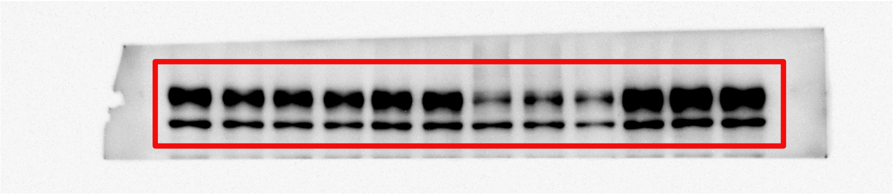

P-AKT

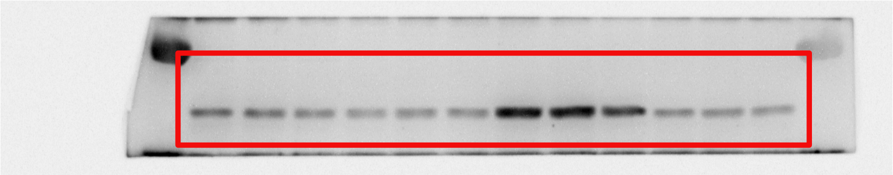

AKT

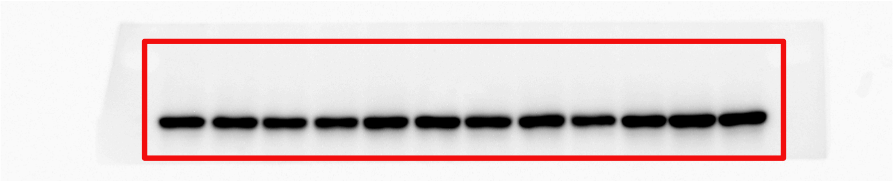

P-ERK

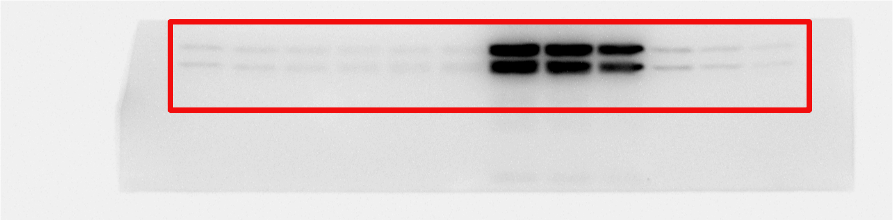

ERK

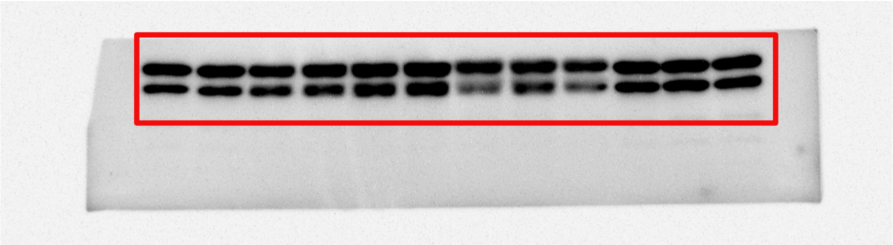

Actin

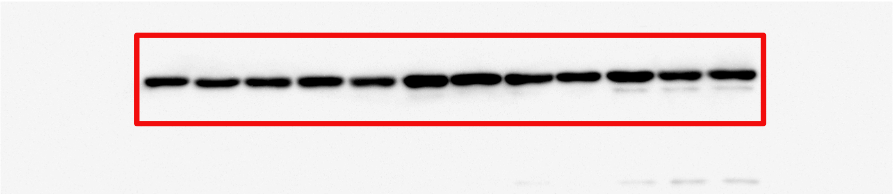

Full unedited blots for Figure 2B

P-CSF1R

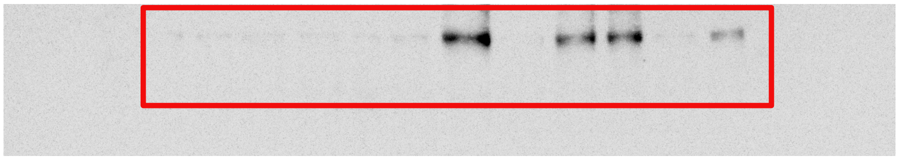

CSF1R

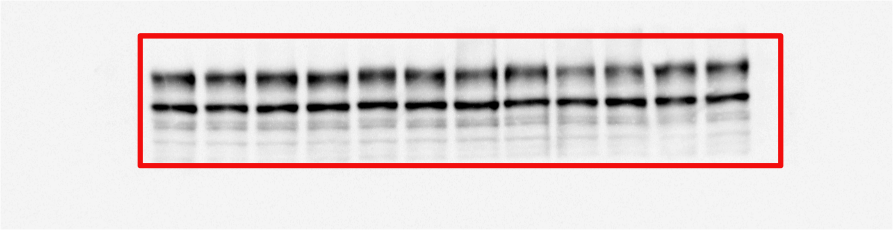

P-AKT

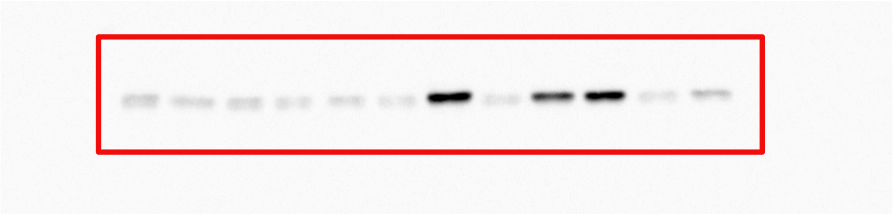

AKT

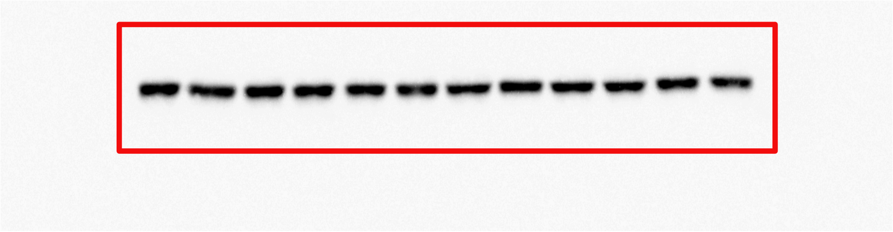

Actin

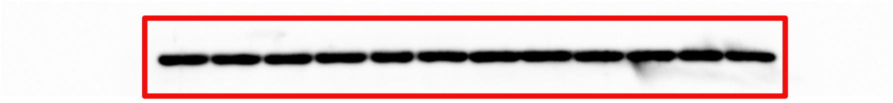

Full unedited blots for Figure 3B

* Membrane images are displayed due to the small size of the membranes.

P-CSF1R

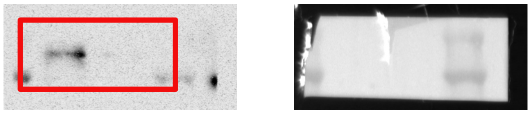

CSF1R

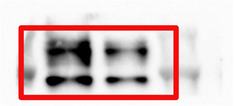

P-AKT

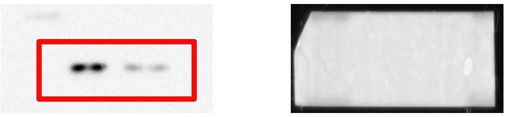

AKT

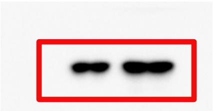

SOCS1

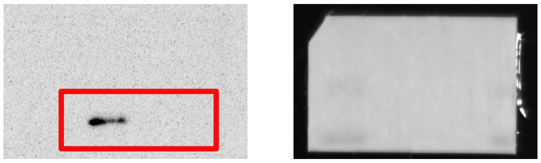

Actin

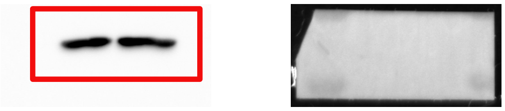

P-STAT1

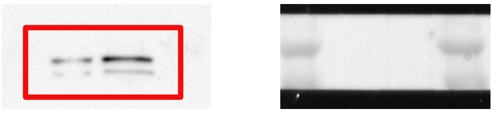

STAT1

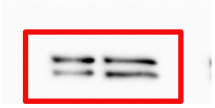

P-STAT3

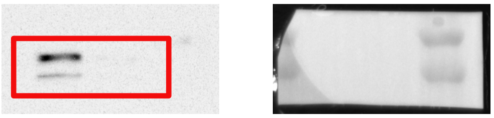

STAT3

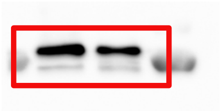

P-NFκB

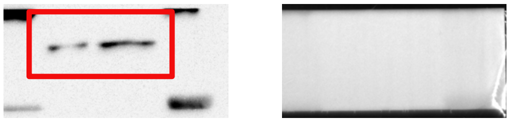

NFκB

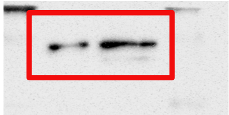

Full unedited blots for Supplemental Figure 3A

P-CSF1R

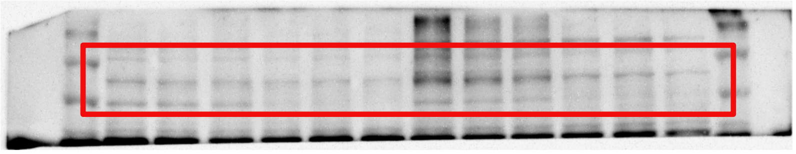

CSF1R

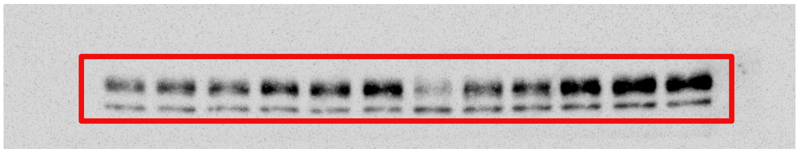

P-AKT

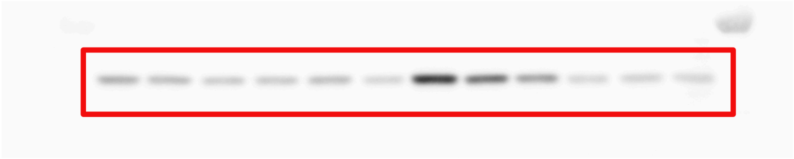

AKT

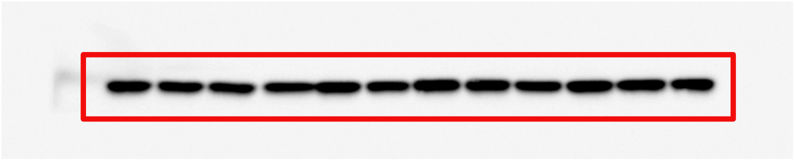

P-ERK

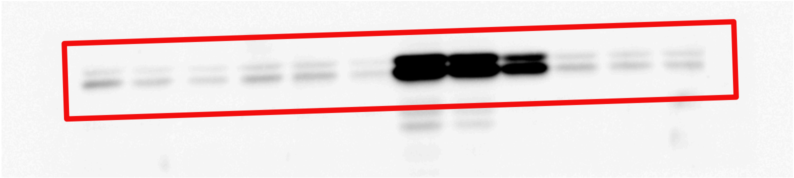

ERK

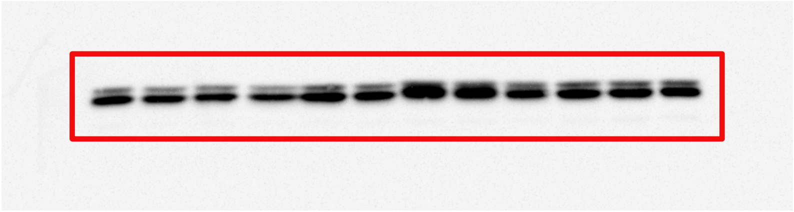

Actin

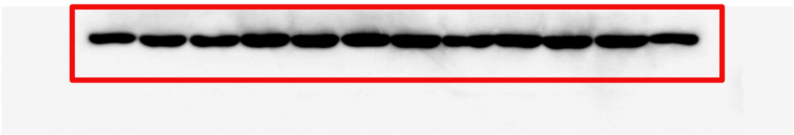

Full unedited blots for Supplemental Figure 3B

P-CSF1R

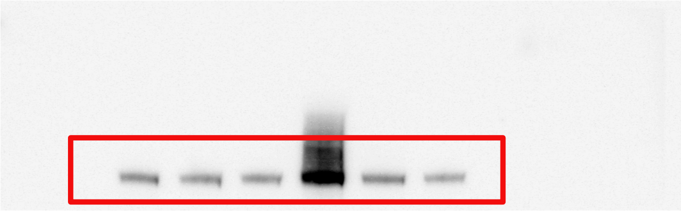

CSF1R

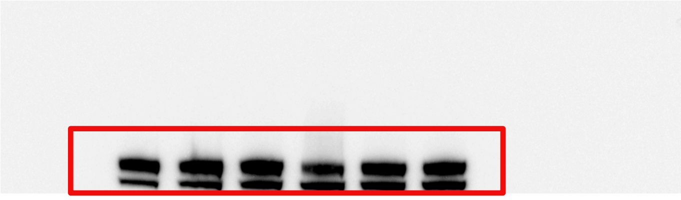

P-AKT

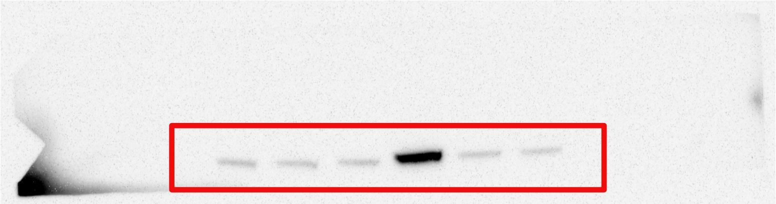

AKT

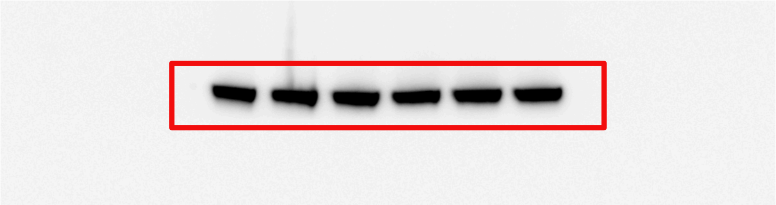

P-ERK

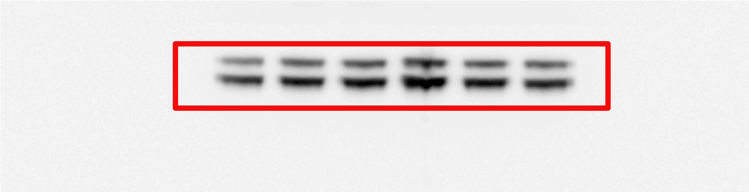

ERK

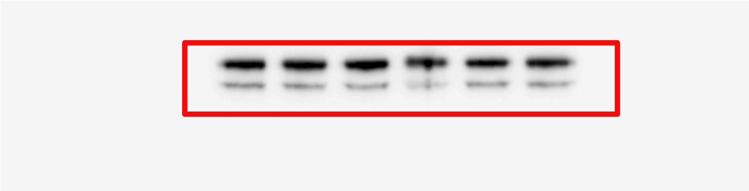

Actin

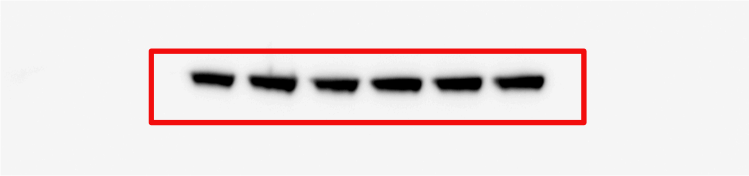

Full unedited blots for Supplemental Figure 6

MCA205

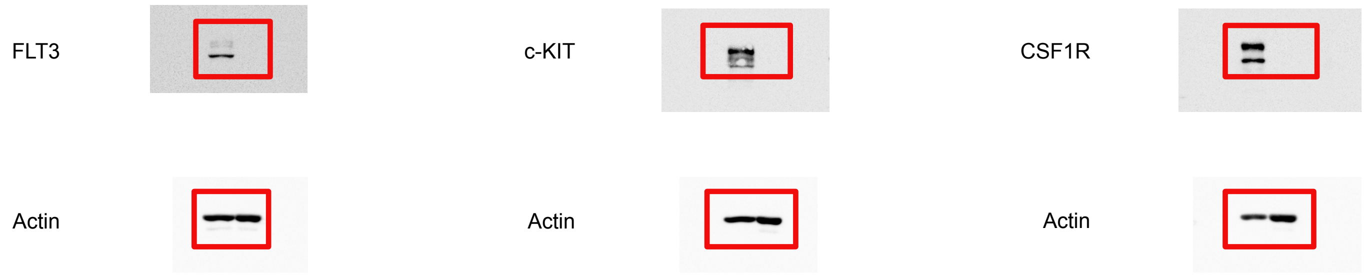

MC38

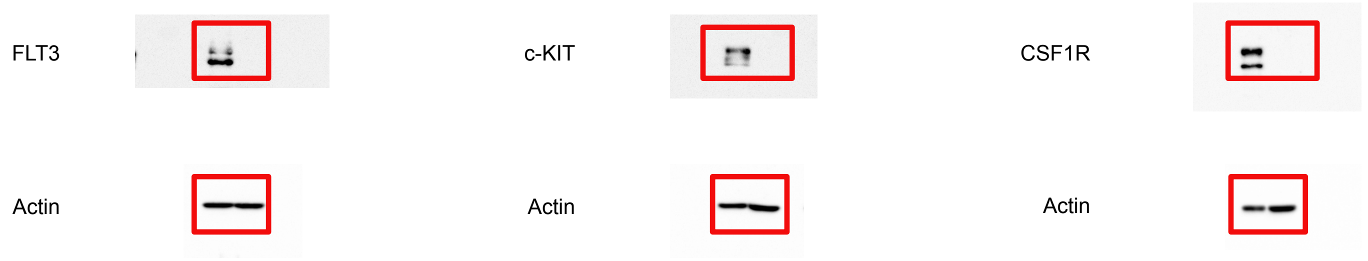

EMT6

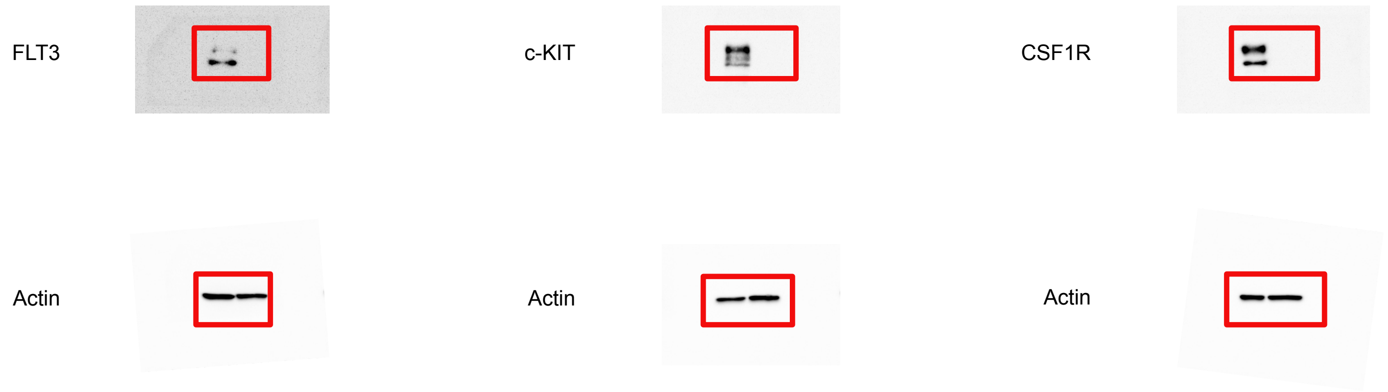

Full unedited blots for Supplemental Figure 7

P-FLT3

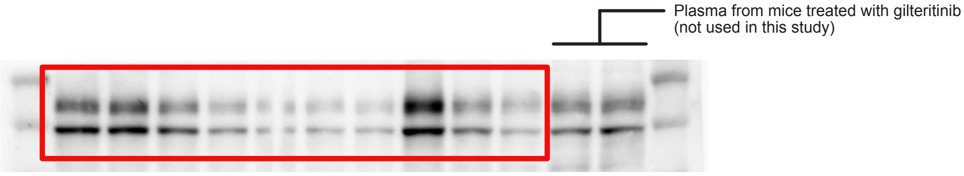

FLT3

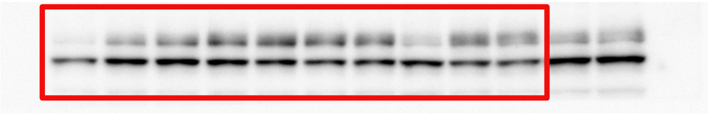

Actin

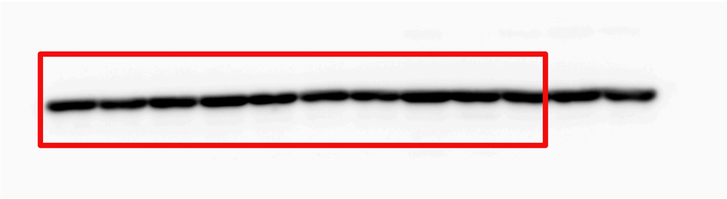

Supplement: Unedited blot and gel images [file jciinsight-10-178146-s047.pdf]
